# Consistent use of lipid lowering therapy in HIV infection is associated with low mortality

**DOI:** 10.1186/s12879-021-05787-4

**Published:** 2021-02-05

**Authors:** Henning Drechsler, Colby Ayers, James Cutrell, Reuben Arasaratnam, Roger Bedimo

**Affiliations:** 1 VA North Texas Health Care System, Dallas, TX USA; 2 UT Southwestern Medical Center School of Medicine, Dallas, TX USA

## Abstract

**Background:**

In people living with HIV (PLWH), statins may be disproportionately effective but remain underutilized. A large prospective trial in patients with low to moderate cardiovascular (ASCVD) risk will reveal whether they should be considered in all PLWH. But its effect size may not apply to real-world PLWH with higher ASCVD and mortality risk. Also, the clinical role of non-statin lipid-lowering therapy (LLT) and LLT adherence in this population is unknown.

**Methods:**

Comparative multi-level marginal structural model for all-cause mortality examining four time-updated exposure levels to LLT, antihypertensives, and aspirin in a virtual cohort of older PLWH. Incident coronary, cerebrovascular, and overall ASCVD events, serious infections, and new cancer diagnoses served as explanatory outcomes.

**Results:**

In 23,276 HIV-infected US-veterans who were followed for a median of 5.2 years after virologic suppression overall mortality was 33/1000 patient years: > 3 times higher than in the US population. Use of antihypertensives or aspirin was associated with increased mortality. Past LLT use (> 1 year ago) had no effect on mortality. LLT exposure in the past year was associated with a reduced hazard ratio (HR) of death: 0.59, 95% confidence interval (CI) 0.51–0.69, *p* < 0.0001 for statin containing LLT and 0.71 (CI: 0.54–0.93), *p* = 0.03 for statin-free LLT. For consistent LLT use (> 11/12 past months) the HR of death was 0.48 (CI: 0.35–0.66) for statin-only LLT, 0.34 (CI: 0.23–0.52) for combination LLT, and 0.27 (CI: 0.15–0.48) for statin-free LLT (p < 0.0001 for all). The ASCVD risk in these patients was reduced in similar fashion. Use of statin containing LLT was also associated with reduced infection and cancer risk. Multiple contrasting subgroup analyses yielded comparable results. Confounding is unlikely to be a major contributor to our findings.

**Conclusions:**

In PLWH, ongoing LLT use may lead to substantially lower mortality, but consistent long-term adherence may be required to reduce ASCVD risk. Consistent non-statin LLT may be highly effective and should be studied prospectively.

**Supplementary Information:**

The online version contains supplementary material available at 10.1186/s12879-021-05787-4.

## **Background**

There is a persistent life expectancy gap of 8–9 years between people living with HIV (PLWH) and the general population [1, 2]. Reasons may include an increased risk for atherosclerotic cardiovascular disease (ASCVD) [3, 4], non-AIDS defining cancers [5], osteoporosis, and accelerated liver fibrosis [6] which have been summarized as HIV-associated non-AIDS comorbidity and carry, along with non-AIDS-defining infections, a higher attributable mortality in PLWH [7, 8]. Hyperlipidemia and premature cardiovascular disease in PLWH were first reported in 1997 [9] and 1998 [10] as a presumed side effect of lifesaving antiretroviral therapy (ART). Physicians, staying true to ‘*primum nil nocere*’, have since monitored serum lipid levels and frequently prescribed lipid-lowering therapy (LLT). This practice continued even after it became apparent that rising serum cholesterol after initiation of highly active antiretroviral therapy (HAART) represent a return to pre-infection levels [11] and that HIV infection itself is a key contributor to ASCVD risk [3, 4].

Hydroxymethylglutaryl-coenzyme A reductase inhibitors (statins) are a major tool for ASCVD prevention in the general population. In PLWH, statins have also been shown to exert beneficial immunomodulatory effects, as suggested by decreased cancer incidence [5], progression of liver fibrosis [12], or chance of HIV virologic rebound [13]. Yet only their lipid-lowering efficacy [14] and not their effectiveness to prevent ASCVD events, has been demonstrated. Since statin use has been linked to greater than 50% reductions in all-cause mortality in several HIV cohorts [15–17], an expanded indication could conceivably contribute to bridging of the life expectancy gap. However, other analyses have either failed to show a statin-associated mortality benefit [17, 18] or found it comparable to the general population [19]. Also, large beneficial statin-attributed treatment effects in some observational studies have been identified as the result of methodological flaws [20]. Given this uncertainty about the true extent of their benefit, statins remain substantially underutilized in PLWH [21] (based on the 2013 AHA/ACC Cholesterol treatment guidelines [22]).

The question whether all PLWH should receive statins may be answered by a large multinational trial of pitavastatin in PLWH aged 40–75 years with low to moderate ASCVD risk, scheduled to conclude by 2023 [23, 24]. But its effect size may not apply to real-world patients with higher cardiovascular and all-cause mortality risk for whom a placebo-controlled trial was not ethically or practically feasible. As these patients may already struggle with polypharmacy and poor ART adherence, a clinician’s enthusiasm to promote statins will be best informed by an accurate estimate of the population-specific clinical benefit.

The US Veterans Affairs (VA) HIV Clinical Case Registry (CCR) was a racially diverse virtual cohort of all HIV-infected US-veterans until 2012, based on the VA’s electronic medical records, including its Pharmacy Benefits Management database [25]. VA-pharmacies are the exclusive source for prescription medications for most US veterans and require very low or no medication co-pays. Their detailed inpatient and outpatient prescription and refill records lend themselves to the creation of granular day-to-day medication exposure models. This allowed for a comprehensive analysis of clinical effectiveness of preventive medications.

## **Methods**

### **Patients and follow-up**

We included all HIV-infected US veterans who received care at VA centres from 1996 to 2011 and achieved an undetectable HIV viral load (VL) after starting HAART. Follow-up began at the day of the first undetectable VL (undetectable at any level or quantified < 50 copies/mL) and ended at the earliest occurrence of: death, loss of clinical follow-up for > 13 months, or 1/1/2012 (end of available data). The VA North Texas Health Care System Institutional Review Board approved this study.

### **Outcomes**

The primary outcome was all-cause mortality. Death dates in the CCR were recorded and updated locally and centrally reconciled with VA benefits databases. The sensitivity of this method has been estimated between 91 and 97% [26]. As cause of death was not available, we additionally examined acute ASCVD events (overall, coronary, or cerebrovascular), severe infections, and new cancer diagnoses as explanatory outcomes. Infection and cancer outcomes were derived from the first relevant international classification of diseases (ICD-9) code after enrolment in the medical record excluding infection codes for cellulitis, upper respiratory infections, or cystitis and cancer codes for squamous and basal cell skin cancers. ASCVD-related ICD-9 codes were often administratively added – possibly to justify use of preventive cardiovascular (CV) medications. To minimize differential outcome misclassification, we excluded all ASCVD events without a well-defined day of onset by using an algorithm based on ICD-9 and procedure codes, laboratory values, and neuroimaging dates (see Supplement 1.2, Fig. S1, Tables S1–3).

### **Medication exposure**

We calculated 1-year “percent of days covered” (PDC) [27] for the following CV medications: 1) LLT: statin compounds, 2) non-statins (NS): fibrates, fish oil preparations, ezetemibe, and niacin; 3) Antihypertensives (AHT): angiotensin antagonists, beta blockers, calcium channel blockers, non-loop diuretics, and others; 4) cardiac aspirin (ASA) and also that of all individual ARV agents. We used a day-to-day exposure model that accounted for hospitalizations, early refills, prescription of incompatible drug classes, and prescription of different drugs within the same class (Supplement 2). HAART adherence was defined as 1-year PDC of accepted combinations of ARVs (Supplement 2). All medication PDCs and HAART adherence were updated weekly and at the day of clinical event and binned into mutually exclusive time-updated exposure levels:

1) *consistent exposure*: exposed ≥11/12 past months (> 91% PDC), *2) recent inconsistent exposure*: any exposure in the last year < 11/12 months, *3) remote exposure*: prior use but not during the last year, and *4) never exposed* (reference category).

Within the consistent exposure level, we differentiated between statin-only and statin-free LLT use - defined as either exclusive or no use of statins during the last year - and assigned all other exposures as combination LLT. For consistent AHT exposures, we distinguished between single and combination AHT. For recent and remote exposures, we distinguished between statin-containing and statin-free LLT. We also studied individual statin compounds and drug classes (NS-LLT, AHT) in a separate model of current exposure (supplement).

### **Statistical models**

We considered main effect and clinically relevant 2-way interactions for any parameter that potentially affected both outcome and likelihood of LLT, AHT, or ASA exposure in prediction models for each endpoint and all presented subgroup analyses. These Cox survival models included: individual ARV-PDCs, 1-year HAART adherence, HIV-specific and metabolic laboratory values, vital signs, and comorbidities. Comorbidity status was derived from ICD-9 or procedure codes and/or laboratory values. PDCs and laboratory covariates were calculated from time-weighted, weekly updated running averages over the past year. TDF was the only individual ARV component independently associated with decreased mortality in the predictor models (Table S7). All significant (*p* < 0.05) terms from the predictor models and the categorized frequency of outpatient follow-up were introduced into generalized linear models to generate propensity scores for each exposure level of each CV medication category and endpoint (Tables S6a/b). Each individual propensity score level was stabilized by its relative frequency and truncated at the 5th and 95th percentile (asymmetric truncation) to reduce unmeasured confounding [28]. The final, inverse probability weighted (IPW) survival models controlled for multi-level exposures to the three CV medication classes and also included a censoring weight (for mortality). We used the Benjamini Hochberg method for multiplicity correction of *p*-values for all analyses in the overall population [29].

### **Computing and software**

Data extraction, cleaning, compilation, medication PDCs, and generalized linear models were calculated with SPSS (Versions 23 to 25, IBM Corporation, Armonk, NY) and Microsoft Excel for Windows (Microsoft Corporation, Redmond, WA). The survival models for the predictor selection were calculated at the Texas Advanced Computing Center at the University of Texas in Austin using the survival package [30] of R, Version 3.4 (Foundation for Statistical Computing, Vienna, Austria). For the final survival models, we used the same package using R, Version 3.53.

## **Results**

### **Cohort composition and comorbidity**

We followed 23,276 patients for a median of 5.2 years, inter-quartile range (IQR): 2.5–9.2 years, which amounted to 140,130 patient years (Table 1, Table S8a). Sixty-six percent of follow-up time was spent during sustained (≥1 year) virologic suppression. Comorbidity rates at end of follow-up, were as follows: 56% nicotine use (ever), 27% prevalent ASCVD (baseline 14%), 26% Hepatitis C, 11% congestive heart failure, 10% peripheral vascular disease, 10% chronic kidney disease (estimated glomerular filtration rate < 60 ml/min), 9% liver fibrosis (aspartate aminotransferase-to-platelet ratio index ≥1.5), and 6% diabetes mellitus.

**Table 1:** Demographics, CV medication experience and HAART effectiveness in three different time periods

Baseline characteristics over time (Median (IQR) or %)	1996–2000	2001–2005	2006–2011	Overall
	*n* = 7434	*n* = 7855	*n* = 7987	*n* = 23,276
Age	50 (44–56)	53 (47–60)	56 (48–64)	53 (46–60)
Female	1.9%	2.4%	3.3%	2.5%
Race				
African American	38%	48%	53%	46%
White	34%	39%	37%	37%
Unknown	27%	12%	8%	15%
Smoking	52%	59%	57%	56%
HCV co-infection	34%	27%	19%	26%
CD4 (/mm^3^)	312 (170–486)	312 (176–484)	355 (220–512)	328 (189–496)
VL LOG before HAART	4.2 (3.3–4.9)	4.6 (3.5–5.2)	4.5 (3.5–5.0)	4.4 (3.4–5.0)
Years HIV Diagnosis-VL suppression	2.9 (0.9–5.3)	3.4 (0.9–7.4)	2.9 (0.8–7.9)	3.0 (0.9–6.6)
Prior CV Medication Exposure				
LLT	2%	9%	12%	8%
AHT	17%	25%	30%	24%
ASA	4%	6%	6%	5%
HAART at study inclusion				
None	7%	12%	4%	8%
Unboosted PI, no TDF	76%	19%	3%	32%
Unboosted PI with TDF	0%	3%	2%	2%
Boosted PI, no TDF	5%	17%	13%	12%
Boosted PI with TDF	0%	11%	25%	12%
EFV or INSTI, no TDF	12%	29%	12%	18%
EFV or INSTI with TDF	0%	9%	41%	17%
One-year HAART adherence (Median)	65%	74%	84%	79%
One-year virologic suppression (Mean)	75%	70%	83%	78%

### **Mortality and censoring**

Twenty percent (*n* = 4622) of the cohort died; 40% during hospitalizations at VA facilities. Mortality (33 deaths/1000 patient years) was more than three times higher than for an age, gender, race, and time matched sample of the US-population [31] but improved over time, most pronounced after 2005 and in patients with sustained virologic suppression (Table S8b). Seventy-two percent of deaths occurred in patients with prevalent ASCVD or prior infection or cancer endpoint and 51% in patients without sustained virologic suppression. Sixteen percent of patients (*n* = 3659) were prematurely censored for interruption of care of > 13 months or loss of clinical follow up before January 1st, 2012.

### **Explanatory end points**

Six percent (*n* = 1304) of patients had an acute ASCVD event (896 acute coronary events, 466 acute cerebrovascular events, 58 with both), 28% (*n* = 6618) a serious infection (9% AIDS defining, 21% other serious infection) and 15% (*n* = 3469) a new cancer diagnosis (Tables S2 and S3).

### **Characteristics of CV medication exposures**

Age specific exposure rates to HAART and LLT were correlated with each other and with virologic suppression and changed over time (Table S8b). Forty-two percent of patients ever took LLT (36% statins, 21% NS-LLT, 15% both), 63% AHT, and 35% cardiac aspirin. Consistent LLT users were co-exposed to AHT for 59% and to ASA for 19% of follow-up time. Persistence of exposure after initial prescription was as follows: 54% of follow-up time for statins, 45% for NS-LLT, 63% for AHT, and 30% for aspirin. The consistent exposure level was characterized by high cumulative drug exposures (median > 4 years, Table S5).

### **Model correctness and covariate balance**

**Fig. 1:**
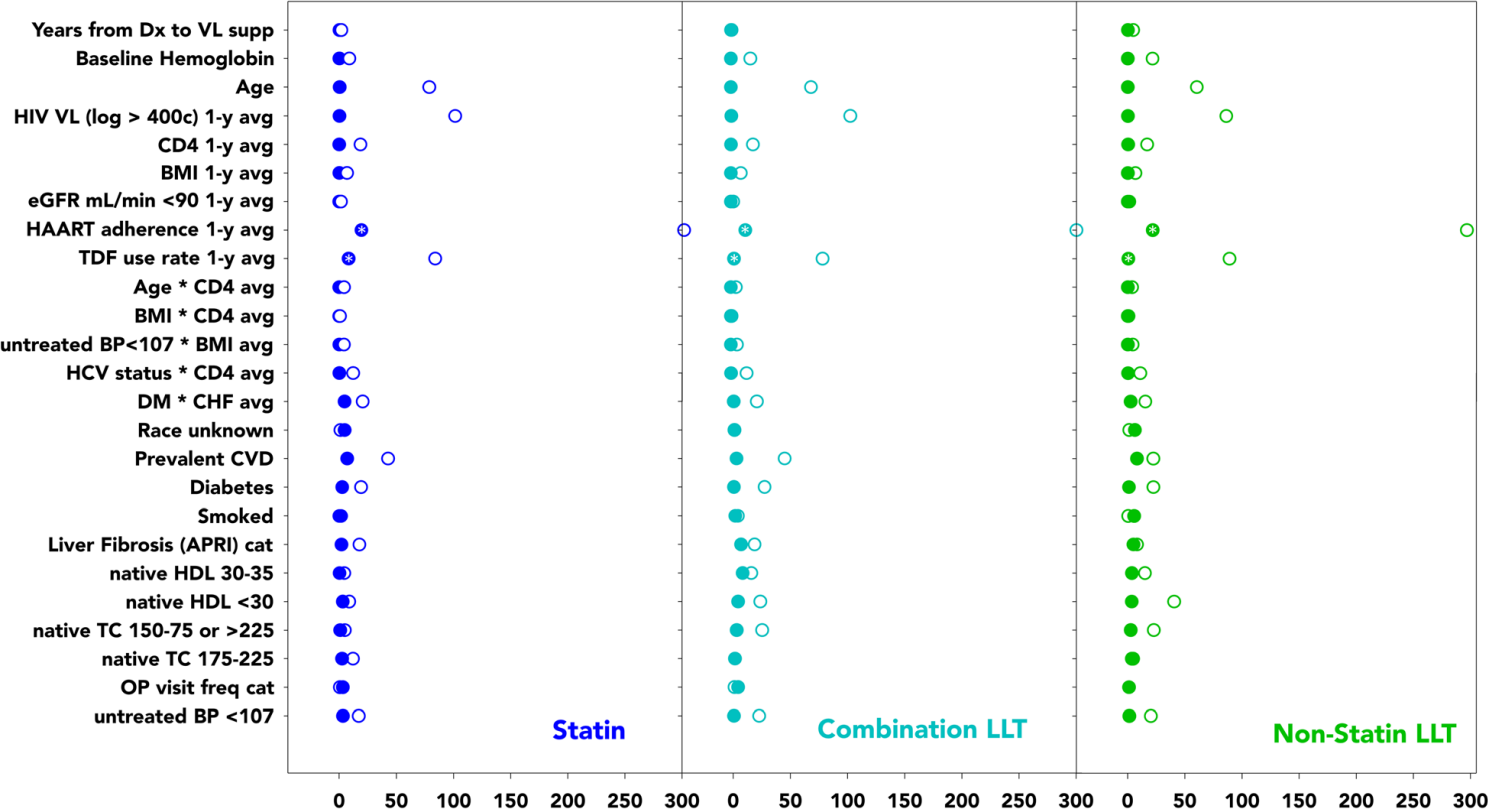
Absolute Standardized Differences between unweighted (empty circles) and weighted (filled circles) covariates for different LLT exposures between consistent users and never exposed patients. Naïve refers to off LLT > 7 days. * relative differences (HAART and TDF use) were negative, i.e. averages were lower after weighting

Figure 1 shows the absolute standardized differences for each of the 25 covariates and interaction terms between consistent LLT users and patients without prior LLT exposure in the mortality model (Fig. S4 for AHT/ASA) and illustrates that covariate balance was achieved [32]. The means of the inverse weights for each exposure level and for each endpoint were almost entirely between 0.9 and 1.1 (except for consistent aspirin use: IPW mean 0.85). We confirmed the proportional hazards assumption by Schoenfeld Residuals. The global impact of weighting and multi-level exposure adjustment is shown in Table S13.

### **All-cause mortality and explanatory outcomes**

Table 2 shows hazard ratios (HR) for all-cause mortality and explanatory outcomes for the different CV medication exposure levels. Exposures to aspirin and antihypertensives (except consistent aspirin use) were associated with increased mortality and ASCVD events, most pronounced for recent inconsistent and least for remote use. When recent inconsistent use was the reference category however, consistent CV medication use was associated with lower mortality and/or risk for ASCVD outcomes (Table S10).

**Table 2:**
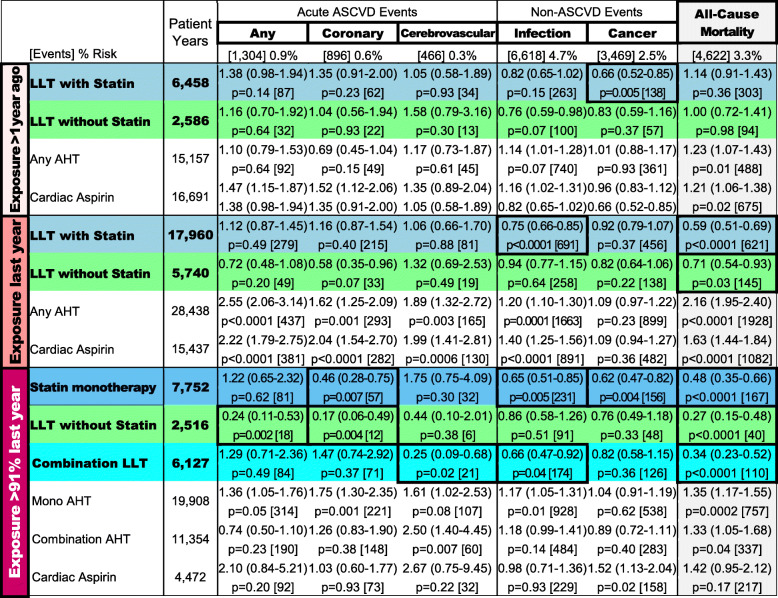
All-cause mortality with explanatory endpoints

Top row of each cell shows hazard ratio (95% confidence interval), bottom row p-value followed by [number of events]. Cells with significant beneficial associations are framed. P-values are multiplicity corrected (Benjamini-Hochberg)

Remote LLT use had no impact on mortality. The mortality risk reduction for patients with recent inconsistent LLT exposures was 41% for statin-containing, and 29% for statin-free LLT. Consistent LLT use was associated with a mortality benefit of 52% for statin-only LLT, 66% for combination-LLT, and 73% for statin-free LLT. Only consistent LLT exposures were associated with a reduced risk for acute ASCVD events but a reduced risk for non-ASCVD outcomes was seen for all statin exposures, including recent (infections) or remote use (cancer).

### **Mortality subgroup analyses**

Table 3 displays the HR for all-cause mortality in contrasting subgroups of patients. The impact of consistent LLT exposure on mortality reduction was most pronounced for patients with incomplete viral suppression and those not receiving tenofovir disoproxil fumarate (TDF) as part of their HAART regimen but was attenuated for TDF users. Consistent use of combination LLT was associated with significantly reduced mortality in almost all examined subgroups which included patients on both sides of the ASCVD risk spectrum. We saw similar mortality risk reductions for patients with high HAART adherence or high use rates of contemporary HAART, particularly for statin-free and combination LLT (Table S12).

**Table 3:**
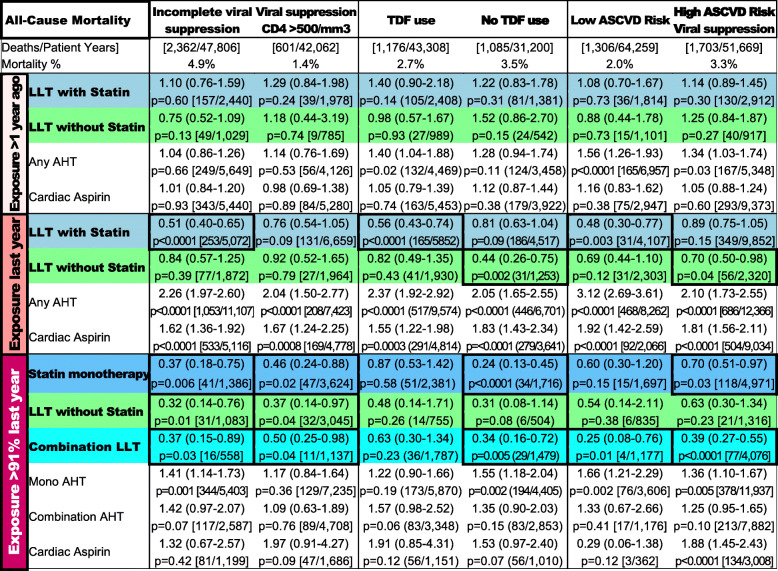
Subgroup analyses. Criteria refer to preceding year

Top row of each cell shows hazard ratio (95% confidence interval), bottom row p-value followed by [deaths/patient years]. Cells with significant beneficial associations are framed (no multiplicity correction). TDF Use: in patients enrolled ≥ 2001. Low ASCVD risk: no known ASCVD, 10-year PCE risk score < 10%, and native LDL cholesterol < 4.1 mmol/L. High ASCVD risk: either known ASCVD, 10-year PCE risk score >= 10%, or native LDL cholesterol >= 4.1 mmol/L

### **Additional analyses**

**Fig. 2:**
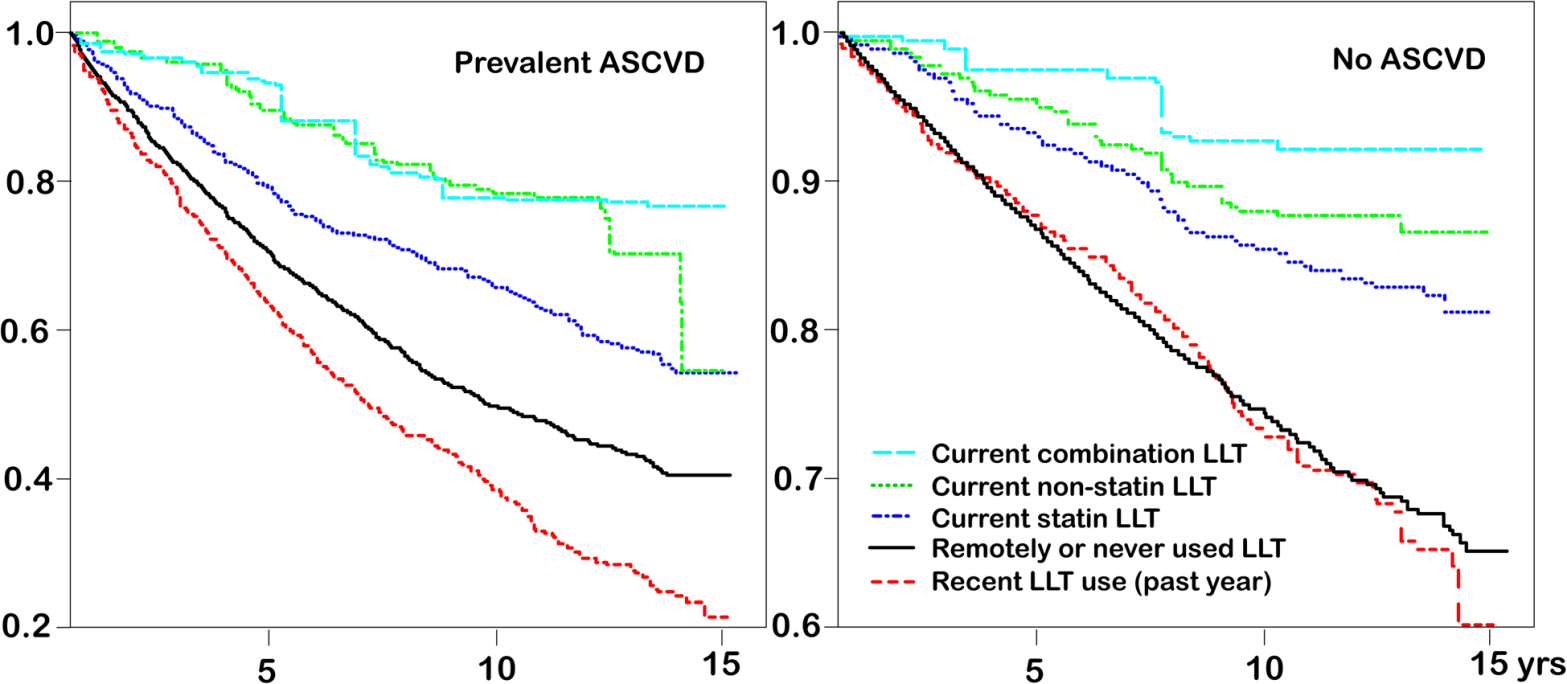
Weighted Survival Curves (all-cause mortality) for *ongoing current* LLT use (≥3/4 last weeks) by ASCVD status. X-axis: Years after initial HIV suppression, Y-axis: proportion surviving

We analysed individual compounds and drug classes in separate models of ongoing use (≥3/4 past weeks), using remotely or never exposed patients as reference. All LLT components but none of the five AHT classes or aspirin were associated with increased survival which was significant for pravastatin, simvastatin, atorvastatin, fibrates, and niacin (Table S9a/b). Figure 2 shows the impact of ongoing current exposure to different LLT exposure levels on all-cause mortality after weighting, stratified by ASCVD status.

We explored the role of immortal time bias by replacing the requirement for 11 months of prior exposure in the consistent use level with > 91.5% use after treatment initiation during the first year and saw virtually identical results. The same also applied when we restricted the analysis to the new LLT users (84% started after enrolment).

We also investigated the impact of absolute serum low-density lipoprotein cholesterol (LDL) levels reached during follow-up in multivariable regression models which adjusted for AHT and ASA use and age. Within the same LLT exposure levels the HR for mortality and explanatory outcomes were similar across a wide array of LDL strata (Table S11). Also, there was no significant interaction between average serum LDL levels and long-term LLT use and mortality reduction.

## **Discussion**

Prior HIV cohort analyses have reported a disproportionately large statin-associated mortality benefit of > 50% [15–17] which resembles reports of ≥40% reduced mortality among statin users in other populations with altered immunity [33–35], inherently increased (cardiovascular) mortality risk [36–39], or old age (25% mortality reduction in men > 75 years) [40]. Decreased mortality had never been observed in primary NS-LLT prevention trials but has recently been reported when icosapent-ethyl (fish oil component) or alirocumab (PSK-9 inhibitor) was added to statins in high-risk populations [41, 42].

The relationship between density of longitudinal LLT exposure and clinical effectiveness is incompletely understood. It could hinge on magnitude of cumulative exposure, consistency of exposure, and recency of use. To capture optimal exposures, “consistent use” in our multi-level exposure model required both > 91% adherence for ≥1 year and use within 30 days. To our knowledge, LLT effectiveness has not been analysed this way in high-risk populations.

Still, the magnitude of the mortality benefit during consistent statin-free LLT use was unexpected and sharply contrasted with only moderately reduced mortality risk for inconsistent use – for which no reduced ASCVD risk was observed. Increased intra-individual (visit-to-visit) serum cholesterol variability has recently been identified as an important ASCVD and mortality risk factor [43, 44]. Although not yet biologically understood, this phenomenon could potentially offset beneficial LLT effects in patients with low adherence and may even play a role in randomized controlled trials of LLT. For statins, the mortality difference between consistent and inconsistent use was much smaller. This may reflect their sustained immunomodulatory properties, as evidenced by reduced infection and cancer risk even for inconsistent, respectively remote users.

Multi-level time-updated drug exposure models have been tested [45], can address frailty bias [28], and are not subject to immortal time bias [20, 46]; both of which are known to lead to inflated treatment effects [20, 28]. The lack of a mortality benefit for remote LLT use argues against healthy user bias [47] and the lack of any benefit for consistent antihypertensive or aspirin use against healthy adherer bias [48] as explanations for the apparent mortality benefit of ongoing LLT use. Our mortality model met consistency, positivity, and correctness of weight-generation criteria of marginal structural models [49]. Similar reductions for overall ASCVD risk during consistent statin-free LLT and coronary risk during consistent statin-only LLT provide biologic plausibility for the reduced mortality risk. Yet after IPW and multi-level adjustment, consistent use of antihypertensives and aspirin remained associated with increased mortality and other adverse outcomes which indicates residual indication bias. This “stubborn” residual bias [50] was directed against patients taking CV medications and would have affected statins similarly. As statins are arguably the most important preventive CV drug class, this residual bias may explain why they appeared less effective than statin-free LLT in reducing mortality. Yet, the lack of a cerebrovascular effect during consistent statin monotherapy is also noteworthy.

The current HAART era is characterized by high adherence to single tablet regimens, sustained virologic suppression, and durable immune restoration. We included patients only after achieving virologic suppression but continued to follow them regardless of virologic failure to avoid informative censoring. We further approximated contemporary conditions in contrasting subgroup analyses and observed comparable results. Consistent combination LLT use remained associated with significantly reduced mortality in all examined subgroups including patients with sustained virologic suppression and immune reconstitution and patients with low ASCVD risk. A notable exception were patients taking TDF containing HAART for whom the mortality impact of consistent LLT was attenuated. TDF (but not tenofovir alafenamide fumarate [51]) has well documented lipid lowering properties [52] and was the only ARV component independently associated with reduced mortality. Importantly, it is no longer used in most modern single-tablet HAART regimens.

There was no apparent association between absolute serum LDL levels and clinical outcomes within the same LLT exposure levels. But if the decreasing mortality risk from remote to consistent LLT exposures is interpreted as “dose-response relationship”, our study would fulfil most of the Bradford-Hill criteria [53] for causal inference between LLT use and mortality risk in PLWH. The REPRIEVE trial [23, 24] will provide the ultimate guidance on statin use in PLWH. But as unmeasured or uncontrolled confounding is unlikely a major explanation for our findings, extensive use of lipid lowering therapy in HIV-infected US-veterans - including those not virologically suppressed - could have saved thousands of lives.

The major strengths of our study are its comprehensiveness, its detailed drug exposure models, and its statistical approach. Others include cohort size and diversity, length of follow-up, and the reliance on uniform data collection on exposures and outcomes across the entire US-VA system. Limitations include an extreme male predominance, the lack of differentiation between different daily doses and the absence of cause of death. Before the publication of the 2013 AHA/ACC Cholesterol guidelines [22], non-statin lipid lowering agents were commonly combined with or substituted for statins in order to target risk-specific cholesterol goals [54]. While its remote timeframe is the major limitation of our study, it also allowed a comprehensive and unique analysis of statin and non-statin LLT effectiveness in PLWH.

## **Conclusion**

Our results emphasize the importance of consistency of LLT exposure and strongly support guideline-conforming use of statins in PLWH [55]. Promotion of LLT adherence in PLWH is likely a high yield intervention which should be combined with regular monitoring of serum lipid levels. The utility of non-statin lipid lowering therapy in PLWH should be studied prospectively.

## Supplementary Information

**Additional file 1.**

## Data Availability

The datasets used and/or analysed during the current study are available from the corresponding author on request.

## Abbreviations

PLWH: People living with HIV
ASCVD: Atherosclerotic cardio- and cerebrovascular disease
LLT: Lipid lowering therapy
CI: 95% confidence interval
HR: Hazard ratio
IQR: Inter quartile range
statin: Hydroxymethylglutaryl Coenzyme A reductase inhibitor
AHA/ACC: American Heart Association/American College of Cardiology
ART: Antiretroviral therapy
VA: United States Department of Veterans Affairs
CCR: VA HIV Clinical Case Registry
VL: HIV plasma viral load
HAART: Highly active antiretroviral therapy
PDC: Percentage of days covered
NS: Non-statin (lipid lowering therapy)
AHT: Antihypertensive(s)
ASA: Cardiac aspirin
TDF: Tenofovir disoproxil fumarate
PI: Protease inhibitor
EFV: Efavirenz
INSTI: Raltegravir or elvitegravir
LDL: Low density lipoprotein cholesterol
PCE: Pooled cohort equation to calculate 10 year ASCVD risk
Dx: Diagnosis
avg.: Average
BMI: Body mass index
eGFR: Estimated glomerular filtration rate
BP: Blood pressure (mmHg)
HCV: Hepatitis C
CHF: Congestive heart failure
DM: Diabetes mellitus
CVD: Cardio- or cerebrovascular disease
HDL: High density lipo¬protein cholesterol (mg/dL)
TC: Total cholesterol (mg/dL)
OP: Outpatient
freq: Frequency
