# Supplementary material for: Consistent use of lipid lowering therapy in HIV infection is associated with low mortality
Source: BMC Infect Dis. 2021 Feb 5;21:150. doi: 10.1186/s12879-021-05787-4 (PMC7866454; doi:10.1186/s12879-021-05787-4)
Supplement: Supplementary file 1 — Additional file 1. [file 12879_2021_5787_MOESM1_ESM.docx]

1. **Highly Effective Lipid Lowering Therapy in HIV Infection Requires High Adherence (Supplement)**

# Methods

## Endpoints

### Death

Death dates in the CCR were manually recorded and updated at local VA facilities and centrally reconciled with VA benefits databases. The sensitivity of VA recorded death data has been estimated between 91-97%.1 Of 4,622 deaths, only 6 were recorded with imprecisely (month or year only) while 32 patients had evidence for clinical follow-up after the recorded death date. The death dates for these combined 38 patients were corrected using algorithmic rules, resulting in an adjustment of <30 days in 23 patients and exactly 1 year in 8 patients (all January death dates). Only 1 of these 38 patients (whose death date was adjusted by 1 day) had ongoing long-term exposure to lipid-lowering agents.

### Acute Cardio- and Cerebrovascular Disease (ASCVD) Outcomes

The composition of acute ASCVD outcomes is tabulated in Tables 1a)-1c) below.

#### Acute Coronary Outcomes

Acute coronary outcomes were derived from either ICD-9 codes, any in- and outpatient procedure codes for angiographic or surgical coronary interventions or the ICD-9 code for unstable angina pectoris if accompanied by evidence for any elevation of troponin I or T values within 7 days, ICD-9, any ICD-9 codes for acute myocardial infarction or coronary occlusion unless there was evidence for normal troponin values within the first 3 days afterwards and no elevated troponin values within a 7 day window. Significant troponin or creatinine kinase elevations meeting criteria were also counted as standalone evidence. See Figure S1 below a schematic representation

##### ICD-9 Codes for acute myocardial infarction or coronary occlusion

410.01, 410.11, 410.21, 410.31, 410.41, 410.51, 410.61, 410.71, 410.81, 410.91, 411.81:
unless there was evidence for normal troponin values within the first 3 days afterwards and no elevated troponin within a 7-day window

##### ICD-9 Code for unstable Angina or CPT/ICD9 Code for coronary procedures

Unstable Angina 411.10. Coronary Procedures CPT codes: 36.01, 36.02, 36.04, 36.05, 36.06, 36.07, 36.09, 36.10, 36.11, 36.12, 36.13, 36.14, 36.15, 36.16, 36.19 or any Outpatient ICD9 Procedure Codes for PTCA: if accompanied by evidence for any elevation of troponin I or T values within 7 days

##### Standalone Laboratory Values qualifying for acute coronary events:

***Troponin I:*** 18x upper limit of normal (ULN) or

>0.5 (if missing ULN) or

Result containing ">"

***Troponin T:*** If eGFR≥60: as above

eGFR<60 or missing: 26x ULN or

>1 (if missing ULN) or

Result containing ">"

if no troponin values available:

Creatine Phospho Kinase (CK) > 250 and CK-MB > 5 and CK-MB index >=6


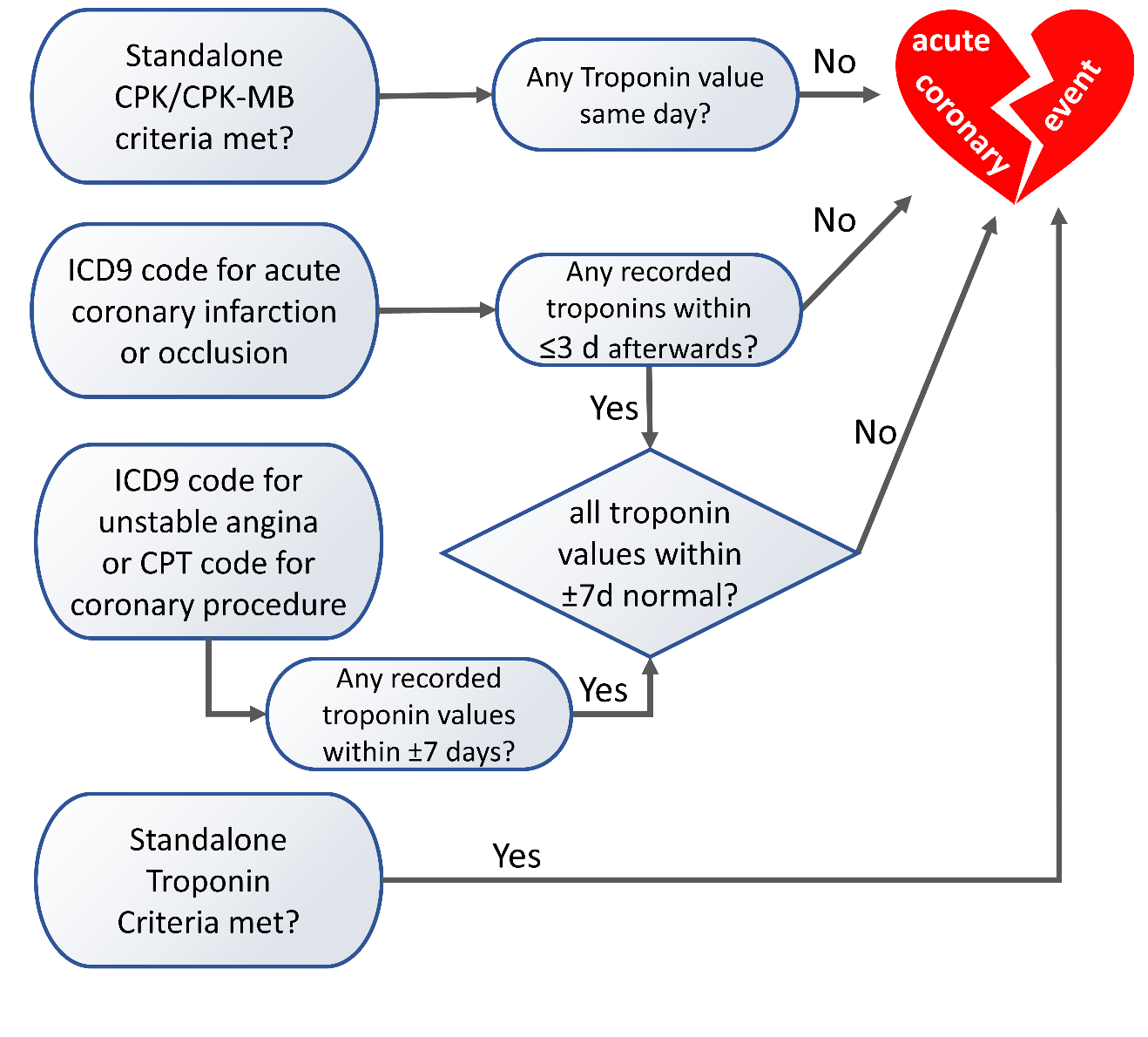


Figure S1: Flow Diagram for Decision Tree for acute coronary events. Abbreviations: d:days

#### Acute Cerebrovascular Outcomes

Acute cerebrovascular outcomes were exclusively derived from these ICD-9 codes: V12.54, 431.00, 433.01, 433.11, 433.21, 433.31, 434.01, 434.11, 434.91, 436.00. In addition, there had to be evidence for neuroimaging (CT, MRI, Angiography) within 7 days of ICD-9 code.

|  | Coronary | | | |  |  | Total |
| --- | --- | --- | --- | --- | --- | --- | --- |
|  |  | Cerebrovascular | | | |  |  |
| Acute Coronary Event | 870 |  | |  | |  |  |
| Acute Events (both) same week |  | 7 | |  | |  | 1304 |
| Acute Cerbrovascular Event |  |  | | 427 | |  |  |
| Acute Coronary after Cerbrovascular Event | 19 |  | |  | |  |  |
| Acute Cerbrovascular after Coronary Event |  |  | | 32 | |  |  |
| Total | 896 | | 466 | | |  |  |

Table S1a Breakdown of acute ASCVD events

| ICD-9 code for unstable angina / coronary intervention AND elevated troponin | 171 | (19%) |
| --- | --- | --- |
| ICD-9 code for AMI / coronary intervention AND no evidence for normal troponin | 480 | (54%) |
| Coronary ICD-9 code / coronary intervention AND CK/CK-MB criteria | 71 | (8%) |
| CK/CK-MB criteria only (standalone) | 67 | (7%) |
| Troponin criteria only (standalone) | 107 | (12%) |

Table S1b Breakdown of acute coronary events. Abbreviations: AMI: acute myocardial infarction, CK: Creatine Kinase

| ICD-9 Codes |  |  | Neuro-Imaging Type |  |
| --- | --- | --- | --- | --- |
| Intracerebral hemorrhage | 7% |  | Conventional or CT angiography | 1% |
| Intracerebral embolism | 3% |  | CT | 70% |
| Intracerebral occlusion with infarct | 39% |  | Conventional MRI | 24% |
| Acute but ill defined ceberovascular disease | 51% |  | MRI angiography | 5% |

Table S1c Breakdown of acute cerebrovascular events

### Infections

The date of the Infection endpoint was set at the as the first appearance of one of the following ICD-9 code during follow-up: 002.0-018.93; 031.0-031.9, 036.1-038.9, 040.0, 046.3-049.9, 052.0, 053.0, 054.3-054.72, 064.0, 078.5,100.81, 112.4-112.84, 114.2, 115.01-115.99, 117.5, 130.0-130.9, 136.3, 320.0-325.0, 421.0-421.9, 424.9, 424.91, 424.99, 480.0-488.12, 507.0, 510.0, 510.9, 513.0, 540.0-540.9, 566.0, 567.0-567.9, 569.5, 572.0, 572.1, 575.0, 590.0-590.9, 595.89, 647.33, 647.34, 682.2, 728.86, 730.0-730.09, 790.7, 995.91, 995.92, 997.31, 997.39.

### Cancer

First appearance of one of the following ICD-9 codes: 140.0-172.9, 174.0-209.73.

| Type of Infection | Frequency |  |  |
| --- | --- | --- | --- |
| Pneumocystosis (PJP) | 480 (7%) | AIDS-related  or -defining 26% | |
| Tuberculosis | 542 (8%) |  |  |
| Candidial esophagitis | 352 (5%) |  |  |
| Cytomegalovirus disease | 113 (2%) |  |  |
| Disseminated/unspecified extrapulmonary NTM disease | 154 (2%) |  |  |
| Cryptococcal meningitis | 72 (1%) |  |  |
| Toxoplasmosis | 69 (1%) |  |  |
|  |  |  |  |
| Pneumonia | 1569 (24%) |  |  |
| Pneumococcal pneumonia | 169 (3%) |  |  |
| Aspiration Pneumonia | 108 (2%) |  |  |
| Bacterial Enteritis | 386 (6%) |  |  |
| Bacteremia | 358 (5%) |  |  |
| Septicemia | 268 (4%) |  |  |
| Infections of kidney | 170 (3%) |  |  |
| Endocarditis | 131 (2%) |  |  |
| Anal or rectal abscess | 108 (2%) |  |  |
| Peritonitis and retroperitoneal infections | 105 (2%) |  |  |
| Abdominal abscess or cellulitis | 101 (2%) |  |  |

| Cancer Type | ICD-9 Codes | Frequency |  |  |
| --- | --- | --- | --- | --- |
| Oropharyngeal | 140-41; 143-9; 161 | 231 (7%) |  | Potentially Infection related 27% |
| Stomach | 151. | 17 (0.5%) |  |  |
| Anal | 154.2-8 | 184 (5%) |  |  |
| Hepatocellular | 155. | 207 (6%) |  |  |
| Kaposi Sarkoma | 176. | 265 (8%) |  |  |
| Female and Male Genitalia | 180-84,187. | 49 (1%) |  |  |
| Bladder | 188. | 96 (3%) |  |  |
| Non Hodgkin Lymphoma | 200,202 | 289 (8%) |  |  |
| Colorectal | 153-154.1 | 219 (6%) |  |  |
| Pancreas | 157. | 38 (1%) |  |  |
| Bronchial | 162. | 358 (10%) |  |  |
| Malignant Melanoma | 172. | 90 (3%) |  |  |
| Prostate | 185. | 445 (13%) |  |  |
| Renal | 189. | 97 (3%) |  |  |
| Hodgkin's Lymphoma | 201. | 65 (2%) |  |  |
| Leukemias | 204-208 | 71 (2%) |  |  |
| Salivary Glands |  | 76 (2%) |  |  |
| Head, Face, and Neck |  | 71 (2%) |  |  |
| Lymph nodes |  | 42 (1%) |  |  |
| Fibromysarcoma |  | 32 (1%) |  |  |
| Unknown Primary / Metastatic Disease |  | 232 (7%) |  |  |

Table S2 and S3: Breakdown of most common cancers (Frequency ≥1%) and infections (Frequency ≥2%)

## Medication Exposures

### PDC Generation

Based on the assumption that patients were continuously exposed to filled outpatient medications until they ran out of drug supply, we tabulated uninterrupted exposure episodes on a day-to-day basis for different ARV categories and overall HAART adherence, statins, NS-LLT, antihypertensives, and cardio-protective aspirin.

Using outpatient fill and refill data (number of days supplied) as well as inpatient prescriptions, we tabulated exposure episodes for antiretrovirals (ARVs), statins, and different classes of cardio­vascular preventive medications (CVPMs). We calculated day-to-day exposure episodes for individual medications or fixed dose combination (FDC) of the following classes of medications:

ARVs: 34 medications (7 FDCs): 28 compounds

Statins: 7 medications (1 FDC): 6 compounds

Antihypertensives (AHT): 64 medications (17 FDCs): 47 compounds

Non-Statin lipid-lowering agents (ALP): 18 medications (1 FDC):10 compounds

Cardiac Aspirin (ASA): 1 compound. To exclude aspirin, use for pain or fever we excluded all doses >325mg, and any prescriptions containing terms like ‘fever‘, ‘pain’, ‘ache’, ‘as needed’, or “PRN’ in the signature.

As instructions to patients how to take the prescribed medications were not available for review, we assessed whether the prescription of a new medication was likely to result in the discontinuation of another medication of the same class. For example, the prescription of an FDC was deemed to terminate the previous exposure to any of its component previously taken as mono-substance or another FDC. Similarly, the prescription of a different statin was deemed to override a previously issued statin prescription. We thus divided all medication classes (except aspirin) into subclasses and modeled concurrent fills within the same subclass as consecutive exposures. Table S1 below lists the subclasses used to differentiate between consecutive and concurrent exposures and Figure S1 illustrates the principles of the translation process. We defined the start date of any exposure as: a) day of fill for outpatient fill at a VA facility (window fill) or inpatient prescription, b) 3^rd^ day after mail order fill. We defined the stop date (first day of non-exposure) as either: a) day after hospitalization without corresponding inpatient prescription, b) day of outpatient fill of a competing drug within the same subclass, or c) the day after the accumulated drug supply was exhausted, taking into account the accumulated drug supply resulting from early refills and hospitalizations. During hospitalizations, only inpatient prescriptions were counted towards exposure, but medication exposure was complemented with inpatient prescription data. This process is analogous to a recently proposed method for estimation of time-varying drug adherence.^2^ For statins and ARVs, we allowed for drug recycling: We defined ‘spare supply’ resulting from early discontinuation after prescription of another medication of the same subclass and counted it towards overall drug supply for the next exposure episode if the original medication was refilled or re-prescribed within one year. Finally, to determine overall class exposure, we translated exposure to fixed dose combinations into individual compounds and then merged exposures back into subclasses (AHT or LLT only). For each week after the initial virologic suppression, we determined Proportion of days covered (PDC) measuring the extent of recent exposure, defined as *p*roportion of *d*ays *c*overed by drug exposure over the past 4 weeks (ongoing exposure model) and past year (long-term exposure model).

PDC was calculated by subtracting cumulative medication exposure at days -28 or -365 from the total cumulative medication exposure for each patient and then dividing the result by the corresponding time interval ^3-5^. PDC as an objective measure of medication adherence for a 90 day window for HAART adherence has been shown to accurately predict virologic failure ^6^. We defined HAART as either: ≥1 anchor drug with ≥2 NRTIs, triple-class therapy, a protease inhibitor (PI) with ritonavir (bPI) plus a non-nucleoside reverse transcriptase inhibitor, or triple NRTI therapy if it contained: (tenofovir or abacavir) *and* zidovudine *and* (lamivudine or emtricitabine). In addition to overall HAART 1-year adherence we considered the following ARV categories for our multivariable models: tenofovir, abacavir, lamivudine/ emtricitabine, older nucleoside analogues, protease inhibitors (boosted or unboosted), and efavirenz/integrase inhibitor.

| Antiretrovirals: |
| --- |
| Zidovudine / Stavudine |
| Emtricitabine / Lamivudine |
| NNRTIs |
| Fosamprenavir / Amprenavir |
| All Fixed Dose Combinations (FDC): compound or class overlap between FDCs and/or contained monosubstance |
| **Statins:** All compounds |
| Antihypertensives: |
| 1) Angiotensin Receptor Blockers /  Angiotensin Converting Enzyme Inhibitors |
| 2) Beta Blockers |
| 3) Calcium Channel Blockers |
| 4) Thiazide Diuretics |
| 5) Miscellaneous  (5a-5f each treated as subclass) |
| 5a) Clonidine |
| 5b) Methyldopa |
| 5c) Isosorbide Mono or Dinitrate |
| 5d) Spironolactone |
| 5e) Reserpine |
| 5f) Hydralazine |
| FDC: Any compound or subclass overlap between FDCs and corresponding mono-substance was modeled as consecutive exposure |
| Non-Statin LLT: |
| Fibrates (gemfibrozil and fenofibrate) |
| Fish Oil (all preparations) |
| Ezetemibe |
| Niacin (all preparations) |

Table S4: Drug Subclasses modeled as consecutive exposures


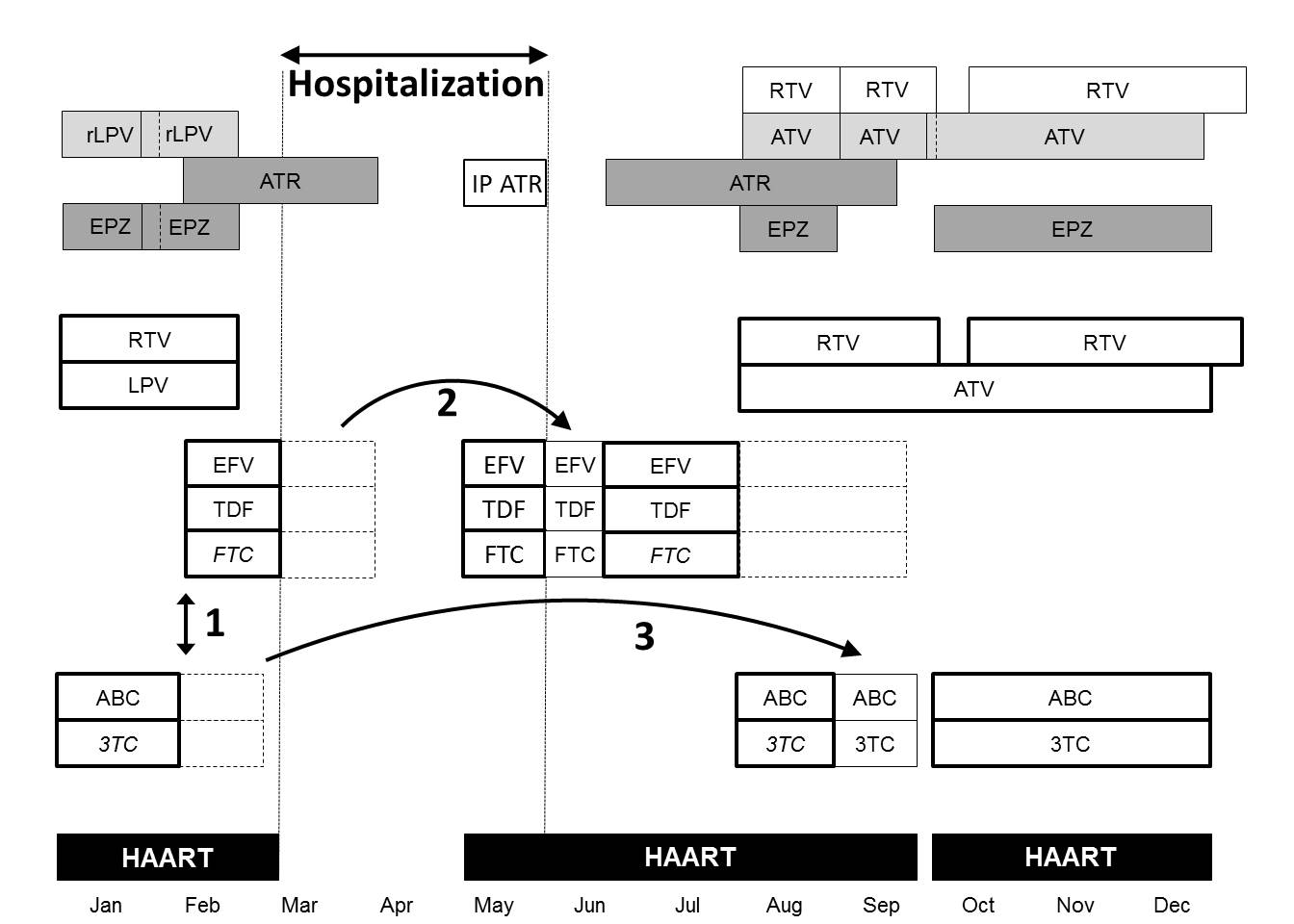


Figure S2: Pharma-Epidemiologic Exposure Model: Translating drug prescriptions and refills into PDCs for different drug categories. Example of HAART adherence in a hypothetical patient. Arrow 1): concurrent exposure modeled as consecutive exposure because of FTC and 3TC belonging to same subclass of drugs (Table S1). Arrow 2): Outpatient supplies during hospitalizations are counted towards post-hospitlization exposure. Arrow 3): Outpatient excess/unused supplies resulting from a competing newer prescription were counted towards overall supply if the same drug was re-prescribed within 1 year (recycling). Abbreviations: IP=Inpatient, rLPV: Fixed dose combination (FDC) of ritonavir (RTV) and Lopinavir (LPV), ATR: FDC of efavirenz (EFV), emtricitabine (FTC), and tenofovir (TDF); EPZ: FDC of abacavir (ABC), lavmivudine (3TC);ATV: atazanavir.

### Cumulative For Different E

| (Ongoing Long-Term Exposure Model) | LLT | Statins | NS-LLT | AHT | Combo AHT | ASA |
| --- | --- | --- | --- | --- | --- | --- |
| Remote Exposure | 0.9 (0.3-2.1) | 0.4 (0.0-1.5) | 0.0 (0.0-0.6) | 0.5 (0.1-2.0) | 0.0 (0.0-0.1) | 0.5 (0.1-1.4) |
| Recent Exposure | 2.2 (0.9-4.6) | 1.5 (0.3-3.6) | 0.0 (0.0-1.2) | 2.6 (0.8-5.4) | 0.2 (0.0-1.8) | 1.5 (0.4-3.4) |
| Consistent Exposure: Statins only | 5.2 (2.8-7.7) | 4.9 (2.7-7.5) | 0.0 (0.0-0.0) |  |  |  |
| Consistent Exposure: NS-LLT only | 5.1 (3.0-8.3) | 0.0 (0.0-1.4) | 4.6 (2.8-7.4) |  |  |  |
| Consistent Exposure: Combination LLT | 7.3 (4.6-9.9) | 5.5 (2.9-8.3) | 4.7 (2.4-7.2) |  |  |  |
| Consistent Exposure: Any AHT |  |  |  | 5.9 (3.4-8.8) | 1.0 (0.0-3.5) |  |
| Consistent Exposure: Combination AHT |  |  |  | 8.0 (5.3-11) | 5.3 (3.2-8.2) |  |
| Consistent Exposure: ASA |  |  |  |  |  | 4.2 (2.5-6.9) |

Table S5: Median years of cumulative use (inter quartile range) at the end of follow-up for the different exposure categories of the ongoing long-term use model.

## Other Covariates

### ICD-9 codes

We defined substance abuse at the time of virologic suppression as the presence of ≥ 2 indicative ICD-9 codes. Smoking status (ever smoked) was handled as a time-dependent variable and its onset was set of either the date of the first qualifying ICD-9 code or the date of the first prescription for varenicline or nicotine substitution products.

### Vital Signs

We used the first systolic blood pressure measurement of every week to calculate time-updated 1-year running blood pressure averages. Days with recorded blood pressure measurements outside of hospitalizations were also used as a surrogate marker for outpatient follow-up frequency which was used in the propensity score models.

Body weight and height was used to calculate BMI and height was also used to calculate body surface area to normalize the calculated creatinine clearance (see below).

### Laboratory, Comorbidity, and Health Care Utilization Covariates

We used VA National Laboratory Test codes and Logical Observation Identifier codes and custom text string searches to identify all relevant laboratory data. All laboratory values were handled as ‘last value carried forward’ using the last value of each week of follow up and then calculating a time weighted running average for the past 52 weeks. The same approach was applied to HIV viral rebound (log/mL above 400 cop/mL), CD4 count, APRI score^7^ (liver fibrosis), estimated glomerular filtration rate (CKD Epi formula^8^, normalized by body surface area), body mass index (BMI) and blood pressure measure­ments. For serum total cholesterol (TC) and high-density lipoprotein (HDL) cholesterol values we only included values if the patient had been off lipid-lowering therapy >7 days. For diabetes we used a composite of either/or ICD-9 code or any glucose value>11.1 mmol/L or Hemoglobin A1c ≥6.5%. For HCV we relied on serologic or PCR tests only, for peripheral vascular disease, heart failure, drug use on ICD-9 diagnosis codes only. Time-updated HCV status was deemed positive if there was HCV antibody test without evidence for undetectable HCV-RNA and became negative once an undetectable HCV RNA test was recorded. To determine prevalent CVD status, we used both ICD-9 and procedural codes (surgical or angiographic coronary interventions). Time updated smoking status was defined by either ICD-9 code or initial prescription of a nicotine substitute or varenicline. VA station size was calculated from the number of unique prescribers of antiretrovirals per calendar year, outpatient follow-up frequency by the number of outpatient blood-pressure measurements during the last 12 months.

## Statistical Models

Table S6 below contains all significant covariates, cofactors, and their interaction terms from the outcome predictor models that were used for the propensity score models for the consistent use models (main analysis).

For individual statin compounds, ezetimibe, niacin, fibrates (gemfibrozil or fenofibrate), and fish oil compounds, as well as the five AHT categories (angiotensin antagonists, beta blockers, calcium channel blockers, non-loop diuretics, and others), and aspirin we used exposure models based on 1-month PDCs of current (ongoing) medication use (see below). We separated between patients with and without prevalent ASCVD, both for the predictor variable generating Cox models and for the propensity score generating generalized linear models.

Current Exposure Model (individual compounds and survival curve)

1) Current (ongoing) exposure: exposed ≥3/4 past weeks

2) Recent Exposure: any exposure in the past year not meeting above criteria

3) Remotely or never exposed: last use >1 year ago or never (Reference category)

|  |  | Acute  ASCVD  Event | Acute Coronary Event | Cerebro-vascular Event | Severe Infection | Cancer | All-cause Mortality |
| --- | --- | --- | --- | --- | --- | --- | --- |
| Cofactors | Prevalent ASCVD |  |  |  |  |  |  |
|  | Baseline Heart Failure |  |  |  |  |  |  |
|  | Baseline Peripheral Vascular Disease |  |  |  |  |  |  |
|  | Diabetes mellitus |  |  |  |  |  |  |
|  | Drug Use |  |  |  |  |  |  |
|  | Hepatitis C |  |  |  |  |  |  |
|  | Liver Fibrosis Score (APRI) category |  |  |  |  |  |  |
|  | Smoking Ever |  |  |  |  |  |  |
|  | Anemia (Hemoglobin<13.1 g/dL) |  |  |  |  |  |  |
|  | systolic Blood Pressure category |  |  |  |  |  |  |
|  | native total cholesterol category |  |  |  |  |  |  |
|  | native HDL cholesterol category |  |  |  |  |  |  |
|  | Gender |  |  |  |  |  |  |
|  | Race not documented |  |  |  |  |  |  |
|  | Race white |  |  |  |  |  |  |
|  | Outpatient Follow-up frequency category |  |  |  |  |  |  |
|  | Patients/year in treating facility |  |  |  |  |  |  |
|  | Cancer |  |  |  |  |  |  |
| Covariates | Calendar Year of VL suppression |  |  |  |  |  |  |
|  | Years since HIV Diagnosis at baseline |  |  |  |  |  |  |
|  | Age |  |  |  |  |  |  |
|  | Body Mass Index |  |  |  |  |  |  |
|  | HAART PDC last year |  |  |  |  |  |  |
|  | Abacavir PDC last year |  |  |  |  |  |  |
|  | Tenofovir PDC last year |  |  |  |  |  |  |
|  | Zidovudine/Stavudine/Didanosine PDC last year |  |  |  |  |  |  |
|  | boosted Protease inhibitor PDC last year |  |  |  |  |  |  |
|  | Efavirenz or Integrase Inhibitor PDC last year |  |  |  |  |  |  |
|  | CD4 average last year (time weighted) |  |  |  |  |  |  |
|  | HIV VL (log) avg last year (>400 cop/mL) |  |  |  |  |  |  |
|  | estimated GFR (mL/min <90) |  |  |  |  |  |  |
|  | Hemoglobin at baseline |  |  |  |  |  |  |
|  | Time-updated Hemoglobin |  |  |  |  |  |  |
|  | CD4 avg last year x Age |  |  |  |  |  |  |
| Interaction Terms | Diabetes mellitus x Prevalent ASCVD |  |  |  |  |  |  |
|  | Diabetes mellitus x Heart Failure |  |  |  |  |  |  |
|  | Diabetes mellitus x Race |  |  |  |  |  |  |
|  | Diabetes mellitus x Drug Use |  |  |  |  |  |  |
|  | Body Mass Index x syst BP category |  |  |  |  |  |  |
|  | Body Mass Index x CD4 avg last year |  |  |  |  |  |  |
|  | Prevalent ASCVD x HDL <1.42 mmol/L |  |  |  |  |  |  |
|  | Hepatitis C status x CD4 avg last year |  |  |  |  |  |  |
|  | Hepatitis C status x Race |  |  |  |  |  |  |
|  | HIV VL(log) avg last year (>400 cop/mL) x Race |  |  |  |  |  |  |
|  |  |  |  |  |  |  |  |

Table S6a: Covariates, cofactors, and interaction terms used for propensity scores of the main analyses. Abbreviations: (see text) PDC=percent of days covered, avg=average, GFR=glomerular filtration rate, syst. BP= systolic blood pressure, HDL=high density lipoprotein cholesterol, VL=viral load

|  |  | Acute  ASCVD  Event | Acute Coronary Event | Cerebro-vascular Event | Severe Infection | Cancer | All-cause Mortality |
| --- | --- | --- | --- | --- | --- | --- | --- |
| Cofactors | Prevalent ASCVD | Separate models by prevalent ASCVD status | | | | | |
|  | BaselineHeart Failure |  |  |  |  |  |  |
|  | Baseline Peripheral Vascular Disease |  |  |  |  |  |  |
|  | Diabetes mellitus |  |  |  |  |  |  |
|  | Drug Use |  |  |  |  |  |  |
|  | Liver Fibrosis Score (APRI) category |  |  |  |  |  |  |
|  | Smoking Ever |  |  |  |  |  |  |
|  | Anemia (Hemoglobin<13.1 g/dL) |  |  |  |  |  |  |
|  | systolic Blood Pressure category |  |  |  |  |  |  |
|  | native total cholesterol category |  |  |  |  |  |  |
|  | native HDL cholesterol category |  |  |  |  |  |  |
|  | Race not documented |  |  |  |  |  |  |
|  | Outpatient Follow-up frequency category |  |  |  |  |  |  |
|  | Patients/year in treating facility |  |  |  |  |  |  |
|  | Baseline cancer |  |  |  |  |  |  |
| Covariates | Calendar Year of VL suppression |  |  |  |  |  |  |
|  | Years since HIV Diagnosis at baseline |  |  |  |  |  |  |
|  | Age |  |  |  |  |  |  |
|  | Body Mass Index |  |  |  |  |  |  |
|  | HAART PDC last year |  |  |  |  |  |  |
|  | ART interruption PDC last year |  |  |  |  |  |  |
|  | Abacavir PDC last year |  |  |  |  |  |  |
|  | Tenofovir PDC last year |  |  |  |  |  |  |
|  | Zidovudine/Stavudine/Didanosine PDC last year |  |  |  |  |  |  |
|  | boosted Protease inhibitor PDC last year |  |  |  |  |  |  |
|  | CD4 average last year (time weighted) |  |  |  |  |  |  |
|  | HIV VL (log) avg last year (>400 cop/mL) |  |  |  |  |  |  |
|  | estimated GFR (mL/min <90) |  |  |  |  |  |  |
|  | Hemoglobin at baseline |  |  |  |  |  |  |
| Interactions | CD4 avg last year x Age |  |  |  |  |  |  |
|  | Diabetes mellitus x HDL<1.42 mmol/L |  |  |  |  |  |  |
|  | Smoking Ever x syst BP category |  |  |  |  |  |  |
|  | Body Mass Index x syst BP category |  |  |  |  |  |  |
|  | Body Mass Index x CD4 avg last year |  |  |  |  |  |  |

Table S6b: Covariates, cofactors, and interaction terms used for the ongoing use Propensity Scores for individual agents and classes (supplement only). Abbreviations: see text also. PDC=percent of days covered, avg=average, GFR=glomerular filtration rate, syst. BP= systolic blood pressure, HDL=high density lipoprotein cholesterol, VL=viral load

| Parameter | HR (95% CI) | p-value |
| --- | --- | --- |
| Prevalent ASCVD | 1.59 (1.48-1.71) | <0.0001 |
| HAART PDC last year | 0.66 (0.55-0.79) | <0.0001 |
| any ART PDC last year | 0.82 (0.68-0.98) | 0.03 |
| Tenofovir PDC last year* | 0.82 (0.74-0.90) | <0.0001 |
| native total cholesterol <3.9 mmol/L | (ref) |  |
| native total cholesterol 3.9 – 4.5 or > 5.8 mmol/L | 0.81 (0.75-0.87) | <0.0001 |
| native total cholesterol 4.5 – 5.8 mmol/L | 0.75 (0.70-0.82) | <0.0001 |
| native HDL cholesterol >0.9 mmol/L | (ref) |  |
| native HDL cholesterol 0.78-0.9 mmol/L | 1.18 (1.08-1.29) | 0.0004 |
| native HDL cholesterol <0.78 mmol/L | 1.31 (1.21-1.41) | <0.0001 |
| Smoking Ever | 1.36 (1.27-1.45) | <0.0001 |
| Time since HIV Diagnosis at baseline (per year) | 0.98 (0.97-0.99) | <0.0001 |
| Hemoglobin at baseline (per mg/dL) | 0.92 (0.91-0.94) | <0.0001 |
| systolic native Blood Pressure <107 mm Hg | 3.78 (1.66-8.60) | 0.002 |
| Age (per decade) | 1.31 (1.23-1.39) | <0.0001 |
| Race not documented | 3.97 (3.69-4.26) | <0.0001 |
| Body Mass Index | 0.89 (0.87-0.90) | <0.0001 |
| Diabetes mellitus | 2.57 (2.24-2.95) | <0.0001 |
| HIV VL (log) avg last year (>400 cop/mL) | 1.22 (1.16-1.28) | <0.0001 |
| CD4 average last year (per 100/mm^3^) | 0.57 (0.50-0.64) | <0.0001 |
| estimated GFR (per mL/min <90) | 1.01 (1.01-1.01) | <0.0001 |
| Liver Fibrosis Score (APRI) <1.5 | (ref) |  |
| Liver Fibrosis Score (APRI) 1.5-2.5 | 2.15 (1.92-2.41) | <0.0001 |
| Liver Fibrosis Score (APRI) 2.5-4 | 2.99 (2.60-3.43) | <0.0001 |
| Liver Fibrosis Score (APRI) >4 | 5.70 (4.99-6.50) | <0.0001 |
| CD4 avg last year x Age in decades | 1.04 (1.02-1.05) | <0.0001 |
| Body Mass Index x CD4 avg last year (per 100/mm^3^) | 1.01 (1.00-1.01) | <0.0001 |
| Body Mass Index x native syst BP <107 | 0.95 (0.92-0.99) | 0.007 |
| Body Mass Index x time-updated heart failure* | 1.03 (1.03-1.04) | <0.0001 |
| Hepatitis C status x CD4 avg last year (per 100/mm^3^) | 1.06 (1.04-1.08) | <0.0001 |
| Diabetes mellitus x Heart Failure | 0.55 (0.41-0.74) | <0.0001 |

Table S7: Parameters associated with all case mortality, used for propensity scores (except* because of model instability). Abbreviations: (see text) PDC=percent of days covered, avg=average, GFR=glomerular filtration rate, syst. BP= systolic blood pressure, HDL=high density lipoprotein cholesterol, VL=viral load.

*ARV components considered for initial modeling: 1) boosted protease inhibitors, 2) unboosted protease inhibitors, 3) efavirenz or integrase inhibiotors, 4) abacavir 5) lamivudine or emtricitabine, 6) zidovudine, stavudine, or didanosine

# Results

## Patient Characteristics throughout study

| Decade of Life | HIV VL not always suppressed past year | | | HIV VL suppressed entire past year | | |
| --- | --- | --- | --- | --- | --- | --- |
|  | 1996-2000 | 2001-2005 | 2006-2011 | 1996-2000 | 2001-2005 | 2006-2011 |
| 20s |  |  |  | 0.1% | 0.2% | 0.3% |
| 30s | 1% | 1% | 1% | 1% | 2% | 2% |
| 40s | 2% | 4% | 3% | 3% | 8% | 11% |
| 50s | 1% | 4% | 4% | 2% | 9% | 19% |
| 60s | 0.3% | 1% | 1% | 1% | 3% | 10% |
| 70s | 0.1% | 0.2% | 0.2% | 0.2% | 1% | 2% |
| 80s |  |  |  |  |  | 0.2% |

Table S8a: Proportion of follow-up years within each age stratum by virologic suppression status in three different “HAART eras” (categories representing <0.1% of total follow-up time omitted)

| Decade of Life | HIV VL not always suppressed past year | | | | | | HIV VL suppressed entire past year | | | | | |
| --- | --- | --- | --- | --- | --- | --- | --- | --- | --- | --- | --- | --- |
|  | 1996-2000 | | 2001-2005 | | 2006-2011 | | 1996-2000 | | 2001-2005 | | 2006-2011 | |
| 20s |  | | | | | |  | 56% |  | 68% |  | 69% |
|  |  |  |  |  |  |  | *0%* | 0 | *4%* | 0 | *1%* | 0 |
| 30s |  | 57% |  | 47% |  | 43% |  | 60% |  | 71% |  | 76% |
|  | *2%* | 15.5 | *5%* | 18.1 | *6%* | 11.1 | *2%* | 13.6 | *9%* | 7.6 | *9%* | 3.4 |
| 40s |  | 57% |  | 52% |  | 48% |  | 60% |  | 72% |  | 77% |
|  | *4%* | 39.5 | *11%* | 41.2 | *12%* | 27.0 | *4%* | 25.6 | *14%* | 18.2 | *21%* | 9.7 |
| 50s |  | 63% |  | 56% |  | 50% |  | 66% |  | 75% |  | 78% |
|  | *5%* | 51.1 | *16%* | 67.7 | *16%* | 51.5 | *7%* | 39.1 | *21%* | 31.2 | *28%* | 21.9 |
| 60s |  | 66% |  | 62% |  | 57% |  | 68% |  | 79% |  | 82% |
|  | *8%* | 82.8 | *23%* | 90.9 | *26%* | 79.7 | *10%* | 60.7 | *30%* | 49.6 | *41%* | 33.5 |
| 70s |  | 65% |  | 58% |  | 57% |  | 65% |  | 77% |  | 82% |
|  | *9%* | 93.1 | *26%* | 130.2 | *32%* | 119.7 | *12%* | 109.9 | *33%* | 84.5 | *47%* | 55.4 |
| 80s |  |  |  |  |  |  |  |  |  |  |  | 77% |
|  |  |  |  |  |  |  |  |  |  |  | *46%* | 87.7 |

HAART
population PDC

|  | x% |
| --- | --- |
| *y%* | z.z |

LLT population
PDC

mortality
/ 1,000 py

Table S8b: Mortality rates (black squares bottom right) per 1,000 patient years, HAART PDC (bold face top right), and LLT PDC (cursive bottom left) in the past year within different age strata by virologic suppression status in three different “HAART eras” (categories with <0.1% of follow-up years omitted). Virologic suppression defined as: absence of 2 consecutive VL >200 copies/mL or any VL >1000 copies/mL (categories representing <0.1% of total follow-up time omitted)

## Additional Models

| **Current Use Model** (Ref. remotely or never) | | Patient | **Acute ASCVD Event** | | | **Infection** | | | **Cancer** | | | **All-cause Mortality** | | |
| --- | --- | --- | --- | --- | --- | --- | --- | --- | --- | --- | --- | --- | --- | --- |
|  |  | years | Deaths |  |  | Events |  |  | Events |  |  | Deaths |  |  |
| Total | | 140,130 | 1,304 | HR (95% CI) | p | 6,618 | HR (95% CI) | P | 3,469 | HR (95% CI) | p | 4,622 | HR (95% CI) | p |
| Current use | Multi-statin/lovastatin | 522 | 10 | 1.22 (0.52-2.89) | 0.80 | 30 | 0.79 (0.49-1.27) | 0.51 | 8 | 0.62 (0.24-1.61) | 0.51 | 12 | 0.56 (0.21-1.53) | 0.45 |
|  | Pravastatin | 8,833 | 110 | 0.96 (0.67-1.37) | 0.90 | 249 | 0.71 (0.47-1.08) | 0.29 | 194 | 0.95 (0.70-1.29) | 0.86 | 177 | 0.61 (0.46-0.81) | 0.003 |
|  | Simvastatin | 4,715 | 71 | 0.98 (0.61-1.57) | 0.97 | 158 | 0.60 (0.45-0.80) | 0.002 | 109 | 0.74 (0.51-1.09) | 0.31 | 93 | 0.37 (0.26-0.53) | <0.0001 |
|  | Fluvastatin | 2,762 | 40 | 0.80 (0.51-1.26) | 0.52 | 81 | 0.51 (0.35-0.74) | 0.002 | 70 | 0.99 (0.69-1.42) | 0.98 | 48 | 0.61 (0.34-1.07) | 0.28 |
|  | Atorvastatin | 2,035 | 26 | 0.81 (0.39-1.67) | 0.74 | 75 | 0.87 (0.58-1.31) | 0.70 | 58 | 1.42 (0.92-2.19) | 0.29 | 37 | 0.39 (0.25-0.61) | 0.0004 |
|  | Rosuvastatin | 2,913 | 20 | 1.28 (0.33-4.99) | 0.85 | 65 | 0.96 (0.53-1.75) | 0.95 | 58 | 0.61 (0.32-1.16) | 0.31 | 44 | 0.43 (0.22-0.84) | 0.06 |
|  | Fibrate | 7,558 | 74 | 0.81 (0.46-1.41) | 0.66 | 262 | 0.89 (0.68-1.17) | 0.63 | 158 | 0.96 (0.70-1.32) | 0.89 | 98 | 0.44 (0.28-0.70) | 0.002 |
|  | Fish Oil | 1,968 | 26 | 0.76 (0.37-1.57) | 0.66 | 60 | 1.11 (0.58-2.10) | 0.86 | 42 | 0.67 (0.36-1.26) | 0.41 | 29 | 0.72 (0.27-1.91) | 0.70 |
|  | Ezetemibe | 1,017 | 18 | 0.54 (0.23-1.27) | 0.34 | 24 | 1.08 (0.40-2.93) | 0.95 | 26 | 0.59 (0.24-1.47) | 0.45 | 10 | 0.75 (0.19-2.91) | 0.82 |
|  | Niacin | 2,364 | 35 | 0.61 (0.29-1.27) | 0.37 | 67 | 1.30 (0.54-3.15) | 0.73 | 52 | 1.67 (0.91-3.09) | 0.29 | 27 | 0.28 (0.13-0.61) | 0.005 |
|  | Angiotensin Antagonist | 24,982 | 407 | 1.27 (0.96-1.68) | 0.28 | 1129 | 1.08 (0.94-1.24) | 0.47 | 668 | 1.00 (0.86-1.17) | 0.98 | 660 | 0.97 (0.83-1.14) | 0.85 |
|  | Beta Blocker | 17,211 | 349 | 1.27 (0.95-1.70) | 0.29 | 845 | 0.95 (0.81-1.12) | 0.73 | 476 | 1.02 (0.85-1.21) | 0.95 | 765 | 1.45 (1.22-1.71) | 0.0002 |
|  | Calcium Channel Blocker | 12,075 | 198 | 1.17 (0.86-1.60) | 0.51 | 646 | 1.12 (0.91-1.39) | 0.47 | 375 | 1.18 (0.96-1.44) | 0.29 | 436 | 0.95 (0.78-1.16) | 0.77 |
|  | Non-loop Diuretic | 15,540 | 183 | 0.98 (0.66-1.47) | 0.97 | 639 | 1.13 (0.95-1.34) | 0.37 | 389 | 0.93 (0.77-1.14) | 0.70 | 472 | 1.20 (1.00-1.43) | 0.17 |
|  | Other Antihypertensive | 714 | 22 | 3.16 (1.66-6.01) | 0.002 | 57 | 2.24 (1.49-3.38) | 0.001 | 21 | 1.54 (0.88-2.70) | 0.31 | 102 | 3.07 (2.25-4.19) | <0.0001 |
|  | Aspirin | 10,451 | 218 | 1.86 (1.28-2.70) | 0.005 | 561 | 1.17 (0.92-1.49) | 0.37 | 322 | 1.00 (0.81-1.24) | 0.98 | 522 | 1.39 (1.17-1.66) | 0.002 |
|  | (Any Statin*) | 21,780 | 275 | 1.00 (0.75-1.35) | 0.98 | 665 | 0.68 (0.54-0.84) | 0.0006 | 493 | 0.89 (0.74-1.07) | 0.20 | 411 | 0.52 (0.43-0.62) | <0.0001 |
|  | (Any NS-LLT*) | 11,315 | 121 | 0.67 (0.46-0.97) | 0.04 | 369 | 1.00 (0.72-1.39) | 0.98 | 240 | 1.07 (0.79-1.44) | 0.67 | 142 | 0.42 (0.28-0.63) | <0.0001 |
| Recent Use | Statin | 10,059 | 167 | 1.21 (0.94-1.56) | 0.33 | 438 | 0.86 (0.74-1.00) | 0.19 | 236 | 0.95 (0.78-1.16) | 0.76 | 487 | 0.95 (0.79-1.13) | 0.73 |
|  | Non-Statin LLT | 6,359 | 84 | 1.40 (0.81-2.39) | 0.41 | 281 | 1.20 (0.96-1.51) | 0.29 | 144 | 0.86 (0.69-1.08) | 0.37 | 258 | 1.16 (0.95-1.42) | 0.33 |
|  | Any Antihypertensive | 14,518 | 233 | 2.39 (1.77-3.23) | <0.0001 | 896 | 1.25 (1.11-1.41) | 0.002 | 456 | 1.31 (1.14-1.52) | 0.002 | 1,370 | 2.87 (2.58-3.19) | <0.0001 |
|  | Aspirin | 9,458 | 255 | 4.09 (2.95-5.66) | <0.0001 | 559 | 1.18 (1.00-1.38) | 0.17 | 300 | 1.13 (0.94-1.36) | 0.38 | 777 | 1.70 (1.47-1.96) | <0.0001 |

Table S9a: Compiled IPW models for current medication use (i.e. ≥3 / 4 last weeks) for each endpoint, controlling for all exposure levels of each CVPM class. P-values were corrected for multiplicity (Benjamini-Hochberg) except*, Abbreviations: HR: Hazard Ratio, CI: Confidence Interval

| **Acute ASCVD Events**  (Current use, reference never) | | Patient | Events | **Acute Coronary** | | Events | **Acute Cerebrovascular** | |
| --- | --- | --- | --- | --- | --- | --- | --- | --- |
|  |  | Years | 896 | HR (95% CI) | p | 466 | HR (95% CI) | p |
| Current use | Multi-statin / Lovastatin | 522 | 8 | 1.50 (0.52-4.31) | 0.66 | 0 | - | - |
|  | Pravastatin | 8,833 | 87 | 1.06 (0.75-1.50) | 0.88 | 32 | 0.95 (0.47-1.94) | 0.96 |
|  | Simvastatin | 4,715 | 59 | 1.20 (0.77-1.86) | 0.64 | 19 | 0.91 (0.40-2.08) | 0.93 |
|  | Fluvastatin | 2,762 | 32 | 1.10 (0.66-1.82) | 0.87 | 9 | 0.50 (0.19-1.31) | 0.36 |
|  | Atorvastatin | 2,035 | 17 | 0.79 (0.35-1.76) | 0.74 | 12 | 0.63 (0.26-1.51) | 0.49 |
|  | Rosuvastatin | 2,913 | 18 | 2.25 (0.59-8.63) | 0.45 | 5 | 0.19 (0.05-0.80) | 0.08 |
|  | Fibrate | 7,558 | 54 | 0.49 (0.30-0.81) | 0.02 | 24 | 1.27 (0.53-3.08) | 0.77 |
|  | Fish Oil | 1,968 | 24 | 0.67 (0.32-1.39) | 0.47 | 5 | 0.70 (0.13-3.65) | 0.83 |
|  | Ezetemibe | 1,017 | 15 | 0.55 (0.23-1.33) | 0.40 | 7 | 0.42 (0.10-1.70) | 0.43 |
|  | Niacin | 2,364 | 24 | 0.55 (0.22-1.37) | 0.40 | 11 | 0.52 (0.16-1.70) | 0.47 |
|  | Angiotensin Antagonist | 24,982 | 284 | 1.02 (0.70-1.50) | 0.96 | 154 | 1.89 (1.38-2.58) | 0.0006 |
|  | Beta Blocker | 17,211 | 262 | 1.32 (0.93-1.88) | 0.30 | 111 | 1.05 (0.68-1.63) | 0.93 |
|  | Calcium Channel Blocker | 12,075 | 146 | 1.19 (0.79-1.77) | 0.63 | 66 | 1.23 (0.74-2.06) | 0.64 |
|  | Non-loop Diuretic | 15,540 | 128 | 1.02 (0.61-1.71) | 0.98 | 64 | 0.87 (0.53-1.41) | 0.74 |
|  | Other Antihypertensive | 714 | 15 | 2.38 (1.04-5.45) | 0.002 | 7 | 4.11 (1.40-12.0) | 0.04 |
|  | Aspirin | 10,451 | 163 | 1.88 (1.12-3.14) | 0.005 | 79 | 1.80 (1.21-2.70) | 0.02 |
|  | (Any Statin*) | 21,780 | 221 | 1.21 (0.87-1.70) | 0.26 | 77 | 0.77 (0.50-1.18) | 0.23 |
|  | (Any NS-LLT*) | 11,315 | 91 | 0.64 (0.43-0.95) | 0.03 | 38 | 0.92 (0.44-1.92) | 0.83 |
| Recent Use | Statin | 10,059 | 122 | 1.44 (1.02-2.02) | 0.04 | 55 | 1.00 (0.67-1.49) | 1.00 |
|  | Non-Statin LLT | 6,359 | 69 | 1.48 (0.73-3.02) | 0.28 | 20 | 0.93 (0.53-1.65) | 0.81 |
|  | Any Antihypertensive | 14,518 | 154 | 1.97 (1.50-2.60) | <0.0001 | 91 | 2.52 (1.66-3.84) | <0.0001 |
|  | Aspirin | 9,458 | 192 | 2.53 (1.92-3.33) | <0.0001 | 83 | 3.40 (1.86-6.19) | <0.0001 |

Table S9b: as in Table S9a for individual ASCVD endpoints

## Additional Analyses

|  | | Patient  Years | Acute ASCVD Event | | | Non-ASCVD Event | | Death |
| --- | --- | --- | --- | --- | --- | --- | --- | --- |
|  | |  | Any | Coronary | Cerebrovascular | Infection | Cancer | All-cause Mortality |
| [Events] % Risk | | 140,130 | [1,304] 0.9% | [896] 0.6% | [466] 0.3% | [6,618] 4.7% | [3,469] 2.5% | [4,622] 3.3% |
| Never Exposed | Any LLT | 90,990 | 1.01 (0.81-1.27) | 1.02 (0.79-1.33) | 0.91 (0.60-1.36) | 1.24 (1.12-1.38) | 1.12 (0.98-1.27) | 1.58 (1.38-1.81) |
|  |  |  | p=0.90 (674) | p=0.86 (424) | p=0.63 (260) | p<0.0001 (4,810) | p=0.09 (2350) | p<0.0001 (3,142) |
|  | Any AHT | 65,274 | 0.39 (0.32-0.48) | 0.62 (0.48-0.80) | 0.53 (0.37-0.76) | 0.84 (0.77-0.91) | 0.92 (0.82-1.03) | 0.42 (0.38-0.46) |
|  |  |  | p<0.0001 (271) | p<0.0001 (185) | p=0.04 (89) | p=0.29 (2,803) | p=0.27 (1,388) | p<0.0001 (1,112) |
|  | Aspirin | 103,530 | 0.44 (0.36-0.55) | 0.47 (0.36-0.62) | 0.51 (0.36-0.72) | 0.72 (0.65-0.81) | 0.93 (0.80-1.09) | 0.59 (0.52-0.67) |
|  |  |  | p<0.0001 (611) | p<0.0001 (396) | p=0.0001 (221) | p<0.0001 (4,726) | p=0.38 (2,398) | p<0.0001 (2,648) |
| Remote Exposure | Any LLT | 9,044 | 1.20 (0.86-1.69) | 1.21 (0.82-1.79) | 1.03 (0.57-1.85) | 1.02 (0.84-1.24) | 0.82 (0.65-1.02) | 1.66 (1.34-2.06) |
|  |  |  | p=0.28 (119) | p=0.34 (84) | p=0.93 (47) | p=0.85 (363) | p=0.07 (195) | p<0.0001 (397) |
|  | Any AHT | 15,157 | 0.43 (0.31-0.59) | 0.42 (0.28-0.64) | 0.64 (0.42-0.97) | 0.94 (0.83-1.06) | 0.92 (0.79-1.07) | 0.51 (0.45-0.59) |
|  |  |  | p<0.0001 (92) | p=0.43 (49) | p=0.43 (45) | p=0.55 (740) | p=0.59 (361) | p<0.0001 (488) |
|  | Aspirin | 16,691 | 0.68 (0.51-0.90) | 0.75 (0.52-1.09) | 0.68 (0.44-1.07) | 0.84 (0.72-0.99) | 0.90 (0.74-1.09) | 0.67 (0.57-0.79) |
|  |  |  | p=0.007 (220) | p=0.14 (145) | p=0.09 (83) | p=0.03 (772) | p=0.27 (431) | p<0.0001 (675) |
| Exposure >91% last year | Statin monotherapy | 7,752 | 1.23 (0.63-2.42) | 0.46 (0.28-0.78) | 1.60 (0.63-4.08) | 0.82 (0.62-1.07) | 0.69 (0.51-0.94) | 0.74 (0.52-1.04) |
|  |  |  | p=0.54 (81) | p=0.003 (57) | p=0.32 (32) | p=0.15 (231) | p=0.02 (156) | p=0.08 (167) |
|  | LLT without Statin | 2,516 | 0.24 (0.11-0.53) | 0.17 (0.06-0.49) | 0.41 (0.09-1.86) | 1.07 (0.72-1.59) | 0.85 (0.55-1.34) | 0.43 (0.24-0.77) |
|  |  |  | p=0.0004 (18) | p=0.001 (12) | p=0.25 (6) | p=0.74 (91) | p=0.49 (48) | p=0.004 (40) |
|  | **Combination LLT** | 6,127 | 1.30 (0.71-2.39) | 1.48 (0.73-3.00) | 0.23 (0.08-0.63) | 0.83 (0.59-1.16) | 0.92 (0.64-1.31) | 0.55 (0.35-0.86) |
|  |  |  | p=0.39 (84) | p=0.28 (71) | p=0.005 (21) | p=0.28 (174) | p=0.64 (126) | p=0.009 (110) |
|  | Mono AHT | 19,908 | 0.55 (0.43-0.69) | 1.12 (0.85-1.48) | 0.85 (0.57-1.28) | 0.97 (0.86-1.08) | 0.96 (0.84-1.11) | 0.59 (0.52-0.68) |
|  |  |  | p<0.0001 (314) | p=0.43 (221) | p=0.43 (107) | p=0.55 (928) | p=0.59 (538) | p<0.0001 (757) |
|  | Combination AHT | 11,354 | 0.29 (0.20-0.42) | 0.76 (0.53-1.09) | 1.30 (0.75-2.24) | 0.96 (0.80-1.15) | 0.83 (0.66-1.03) | 0.60 (0.48-0.76) |
|  |  |  | p<0.0001 (190) | p=0.14 (148) | p=0.35 (60) | p=0.67 (484) | p=0.10 (283) | p<0.0001 (337) |
|  | Aspirin | 4,472 | 0.95 (0.37-2.41) | 0.54 (0.29-1.01) | 1.36 (0.36-5.15) | 0.73 (0.52-1.01) | 1.39 (1.01-1.91) | 0.87 (0.58-1.31) |
|  |  |  | p=0.91 (92) | p=0.05 (73) | p=0.65 (32) | p=0.06 (229) | p=0.04 (158) | p=0.52 (217) |

Table S10: IPW multi-exposure adjusted models for explanatory endpoints and all-cause mortality *with inconsistent use (past year) as reference.* Significant advantageous associations are framed. No multiplicity correction.Abbrevieations: Pt Yrs: Patient Years.

| Death | **LDL average < 1.81 mmol/L** | | | | **LDL average 1.81-2.56 mmol/L** | | | | **LDL average 2.56-3.36 mmol/L** | | | | **LDL average ≥ 3.36 mmol/L** | | | |
| --- | --- | --- | --- | --- | --- | --- | --- | --- | --- | --- | --- | --- | --- | --- | --- | --- |
|  | Pat yrs | Events | HR (95% CI) | p-value | Pat yrs | Events | HR (95% CI) | p-value | Pat yrs | Events | HR (95% CI) | p-value | Pat yrs | Events | HR (95% CI) | p-value |
| Statin only | 687 | 28 | 0.99 (0.66-1.48) | 0.97 | 2,583 | 44 | 0.75 (0.57-0.99) | 0.04 | 2,861 | 68 | 0.50 (0.37-0.69) | <0.0001 | 1,621 | 27 | 0.61 (0.42-0.90) | 0.01 |
| Combo LLT | 904 | 20 | 0.98 (0.68-1.43) | 0.93 | 1,992 | 28 | 0.45 (0.31-0.66) | <0.0001 | 1,955 | 31 | 0.44 (0.30-0.65) | <0.0001 | 1,276 | 31 | 0.54 (0.34-0.85) | 0.008 |
| NS LLT only | 406 | 7 | 0.76 (0.39-1.49) | 0.42 | 796 | 15 | 0.40 (0.21-0.77) | 0.006 | 796 | 9 | 0.69 (0.41-1.16) | 0.16 | 518 | 9 | 0.54 (0.25-1.15) | 0.11 |
| Recent LLT | 2,156 | 189 | 1.42 (1.14-1.76) | 0.002 | 5,262 | 246 | 1.03 (0.86-1.24) | 0.74 | 7,584 | 211 | 0.93 (0.78-1.10) | 0.37 | 8,699 | 120 | 0.71 (0.59-0.86) | 0.0003 |
| Remote LLT | 1,153 | 80 | 2.51 (1.99-3.16) | <0.0001 | 2,355 | 100 | 1.55 (1.24-1.93) | <0.0001 | 3,119 | 119 | 1.13 (0.90-1.42) | 0.28 | 2,417 | 98 | 1.29 (1.00-1.65) | 0.05 |
| Never LLT | 14,571 | 311 | 2.45 (2.14-2.79) | <0.0001 | 29,289 | 1,091 | 1.42 (1.25-1.62) | <0.0001 | 30,388 | 908 | 1.87 (1.65-2.12) | <0.0001 | 16,742 | 832 | Ref . |  |
| ASCVD Event |  |  |  |  |  |  |  |  |  |  |  |  |  |  |  |  |
| Statin only | 538 | 19 | 0.47 (0.22-1.01) | 0.05 | 2,280 | 27 | 0.49 (0.33-0.74) | 0.0007 | 2,659 | 28 | 0.51 (0.34-0.78) | 0.002 | 1,533 | 7 | 0.73 (0.45-1.19) | 0.21 |
| Combo LLT | 781 | 20 | 0.59 (0.32-1.10) | 0.10 | 1,792 | 23 | 0.74 (0.49-1.11) | 0.14 | 1,837 | 30 | 0.60 (0.38-0.94) | 0.03 | 1,237 | 11 | 0.95 (0.59-1.52) | 0.82 |
| NS LLT only | 385 | 3 | 0.88 (0.40-1.95) | 0.76 | 770 | 4 | 0.37 (0.15-0.90) | 0.03 | 774 | 5 | 0.32 (0.12-0.87) | 0.02 | 491 | 6 | 0.41 (0.13-1.30) | 0.13 |
| Recent LLT | 1,970 | 128 | 0.76 (0.59-0.97) | 0.03 | 4,728 | 97 | 0.70 (0.56-0.88) | 0.002 | 7,082 | 72 | 0.74 (0.59-0.93) | 0.009 | 8,318 | 31 | 0.88 (0.69-1.13) | 0.33 |
| Remote LLT | 1,074 | 29 | 1.13 (0.73-1.76) | 0.58 | 2,197 | 32 | 0.86 (0.59-1.26) | 0.44 | 2,998 | 34 | 0.73 (0.49-1.07) | 0.11 | 2,353 | 24 | 0.92 (0.62-1.38) | 0.68 |
| Never LLT | 14,350 | 126 | 0.73 (0.49-1.08) | 0.12 | 28,948 | 212 | 0.70 (0.53-0.94) | 0.02 | 30,115 | 214 | 0.73 (0.56-0.95) | 0.02 | 16,630 | 122 | Ref… |  |
| Infection |  |  |  |  |  |  |  |  |  |  |  |  |  |  |  |  |
| Statin only | 511 | 45 | 0.47 (0.22-1.01) | 0.05 | 1,984 | 78 | 0.49 (0.33-0.74) | 0.0007 | 2,279 | 77 | 0.51 (0.34-0.78) | 0.002 | 1,348 | 24 | 0.73 (0.45-1.19) | 0.21 |
| Combo LLT | 682 | 33 | 0.59 (0.32-1.10) | 0.10 | 1,533 | 61 | 0.74 (0.49-1.11) | 0.14 | 1,506 | 58 | 0.60 (0.38-0.94) | 0.03 | 1,035 | 25 | 0.95 (0.59-1.52) | 0.82 |
| NS LLT only | 286 | 16 | 0.88 (0.40-1.95) | 0.76 | 623 | 24 | 0.37 (0.15-0.90) | 0.03 | 617 | 29 | 0.32 (0.12-0.87) | 0.02 | 376 | 20 | 0.41 (0.13-1.30) | 0.13 |
| Recent LLT | 1600 | 328 | 0.76 (0.59-0.97) | 0.03 | 4,023 | 284 | 0.70 (0.56-0.88) | 0.002 | 5,960 | 241 | 0.74 (0.59-0.93) | 0.009 | 7,220 | 131 | 0.88 (0.69-1.13) | 0.33 |
| Remote LLT | 792 | 90 | 1.13 (0.73-1.76) | 0.58 | 1,619 | 104 | 0.86 (0.59-1.26) | 0.44 | 2,397 | 108 | 0.73 (0.49-1.07) | 0.11 | 1,938 | 51 | 0.92 (0.62-1.38) | 0.68 |
| Never LLT | 11,032 | 716 | 0.73 (0.49-1.08) | 0.12 | 23,506 | 1,457 | 0.70 (0.53-0.94) | 0.02 | 25,489 | 1,585 | 0.73 (0.56-0.95) | 0.02 | 14,333 | 1,032 | Ref… |  |
| Cancer |  |  |  |  |  |  |  |  |  |  |  |  |  |  |  |  |
| Statin only | 604 | 29 | 0.72 (0.43-1.22) | 0.22 | 2,263 | 59 | 0.74 (0.55-0.99) | 0.04 | 2,508 | 55 | 0.76 (0.57-1.00) | 0.05 | 1,443 | 15 | 0.70 (0.48-1.02) | 0.06 |
| Combo LLT | 757 | 20 | 1.17 (0.79-1.73) | 0.43 | 1,708 | 34 | 0.89 (0.66-1.20) | 0.43 | 1,688 | 48 | 0.66 (0.46-0.94) | 0.02 | 1,128 | 28 | 0.63 (0.40-0.98) | 0.04 |
| NS LLT only | 347 | 8 | 0.69 (0.33-1.44) | 0.32 | 670 | 13 | 1.04 (0.66-1.63) | 0.87 | 706 | 21 | 0.64 (0.37-1.11) | 0.11 | 443 | 7 | 0.67 (0.33-1.35) | 0.26 |
| Recent LLT | 1,908 | 203 | 0.84 (0.62-1.13) | 0.24 | 4,608 | 180 | 1.05 (0.87-1.28) | 0.59 | 6,732 | 153 | 0.89 (0.74-1.07) | 0.22 | 7,899 | 50 | 0.92 (0.77-1.09) | 0.35 |
| Remote LLT | 1,002 | 36 | 0.91 (0.61-1.33) | 0.62 | 2,023 | 68 | 1.04 (0.79-1.36) | 0.78 | 2,685 | 63 | 0.91 (0.71-1.19) | 0.50 | 2,167 | 28 | 0.62 (0.44-0.88) | 0.007 |
| Never LLT | 13,168 | 383 | 1.32 (1.15-1.51) | <0.0001 | 26,939 | 770 | 1.04 (0.92-1.18) | 0.55 | 28,119 | 724 | 1.09 (0.97-1.24) | 0.16 | 15,624 | 468 | Ref… |  |

Table S11: Outcomes in long-term use models stratified by past year LDL cholesterol averages: Death (censoring weight) and and (N)ADC events (unweighted) stratified by preexisting ASCVD, controlling for all CV medications exposure levels and age. No multiplicity correction. Similar/comparable hazard ratios across LDL strata are framed and shaded in gray. Abbreviations: HR: Hazard Ratio, CI: Confidence Interval, Pat yrs: Patient years

| **All-Cause Mortality** | | **No ASCVD** | **Coronary Disease** | **Cerebrovascular Disease** | **HAART use >75%** | **EFV or INSTI Use >75% and TDF Use >75%** |
| --- | --- | --- | --- | --- | --- | --- |
| [Deaths/Patient Years] | | **2.6%** | **6.1%** | **7.7%** | **[1,659/75,275]** | **[234/15,425]** |
| Mortality % | | **[2,854/111,107]** | **[1,409/23,080]** | **[723/9,389]** | **2.2%** | **1.5%** |
| **Remote Exposure (>1 y ago)** | **LLT with Statin** | 1.37 (0.98-1.93) | 0.91 (0.68-1.23) | 1.18 (0.85-1.63) | 1.41 (1.07-1.87) | 0.67 (0.32-1.39) |
|  |  | p=0.07 [130/4,101] | p=0.55 [151/1,945] | p=0.33 [67/754] | p=0.02 [106/3,191] | p=0.28 [12/923] |
|  | **LLT without Statin** | 0.95 (0.68-1.35) | 0.93 (0.49-1.78) | 1.58 (0.63-3.94) | 1.07 (0.65-1.75) | 1.71 (0.54-5.48) |
|  |  | p=0.79 [61/2,128] | p=0.83 [24/368] | p=0.33 [12/151] | p=0.79 [36/1,368] | p=0.36 [8/327] |
|  | Any Antihypertensive | 1.36 (1.16-1.60) | 0.88 (0.57-1.37) | 0.88 (0.55-1.39) | 1.20 (0.93-1.55) | 1.67 (0.84-3.34) |
|  |  | p=0.0001 [342/11,620] | p=0.58 [110/2,608] | p=0.58 [53/1,259] | p=0.16 [165/7,618] | p=0.15 [26/1,459] |
|  | Aspirin | 1.08 (0.90-1.30) | 1.03 (0.81-1.30) | 1.07 (0.81-1.42) | 1.11 (0.89-1.37) | 1.04 (0.60-1.81) |
|  |  | p=0.42 [300/9,021] | p=0.83 [308/6,229] | p=0.62 [145/2,448] | p=0.35 [234/9,210] | p=0.88 [36/2,105] |
| **Any Exposure last year** | **LLT with Statin** | 0.55 (0.45-0.69) | 0.66 (0.55-0.79) | 1.10 (0.87-1.38) | 0.80 (0.65-1.00) | 0.85 (0.50-1.42) |
|  |  | p<0.0001 [192/11,408] | p<0.0001 [367/5,616] | p=0.44 [184/1,950] | p=0.05 [263/10,167] | p=0.53 [41/2,082] |
|  | **LLT without Statin** | 0.62 (0.45-0.86) | 0.60 (0.40-0.90) | 0.95 (0.57-1.58) | 0.81 (0.54-1.20) | 0.74 (0.27-1.99) |
|  |  | p=0.004 [91/4,581] | p=0.01 [41/922] | p=0.83 [24/367] | p=0.29 [66/3,502] | p=0.54 [8/663] |
|  | Any Antihypertensive | 2.74 (2.45-3.05) | 1.55 (1.07-2.25) | 1.62 (1.19-2.20) | 2.04 (1.70-2.46) | 2.52 (1.52-4.17) |
|  |  | p<0.0001 [1,130/20,216] | p=0.02 [649/6,696] | p=0.002 [334/2,531] | p<0.0001 [522/11,562] | p=0.0003 [82/2,454] |
|  | Aspirin | 1.79 (1.51-2.13) | 1.56 (1.27-1.90) | 1.76 (1.38-2.25) | 1.75 (1.44-2.13) | 1.58 (0.90-2.76) |
|  |  | p<0.0001 [337/7,112] | p<0.0001 [616/6,912] | p<0.0001 [319/2,774] | p=<0.0001 [384/8,294] | p=0.11 [52/1,676] |
| **Exposure >91% last year** | **Statin only LLT** | 0.50 (0.33-0.76) | 0.57 (0.39-0.83) | 0.50 (0.30-0.83) | 0.75 (0.53-1.05) | 0.75 (0.38-1.48) |
|  |  | p=0.001 [62/4,681] | p=0.003 [98/2,701] | p=0.008 [30/821] | p=0.10 [132/6,502] | p=0.40 [25/1,606] |
|  | **LLT without Statin** | 0.36 (0.17-0.76) | 0.43 (0.22-0.83) | 0.61 (0.26-1.43) | 0.28 (0.13-0.62) | 0.32 (0.10-1.01) |
|  |  | p=0.007 [17/1,888] | p=0.01 [19/523] | p=0.26 [8/182] | p=0.002 [29/2,143] | p=0.05 [9/440] |
|  | **Combination LLT  NS-containing LLT*** | 0.32 (0.14-0.72) | 0.40 (0.23-0.67) | 0.36 (0.19-0.70) | 0.53 (0.34-0.81) | 0.25 (0.08-0.80) |
|  |  | p=0.005 [42/3,588] | p=0.0006 [58/2,308] | p=0.002 [20/584] | p=0.004 [79/5,245] | p=0.02 [10/1,107] |
|  | Mono AHT | 1.47 (1.25-1.75) | 1.21 (0.83-1.77) | 1.20 (0.86-1.68) | 1.51 (1.23-1.86) | 2.08 (1.18-3.65) |
|  |  | p<0.0001 [343/12,951] | p=0.33 [350/5,891] | p=0.28 [149/2,012] | p<0.0001 [386/13,480] | p=0.01 [53/2,751] |
|  | Combination AHT | 1.54 (1.08-2.19) | 0.98 (0.64-1.50) | 1.01 (0.68-1.52) | 1.26 (0.95-1.68) | 1.37 (0.69-2.68) |
|  |  | p=0.02 [114/6,062] | p=0.94 [193/4,663] | p=0.96 [83/1,508] | p=0.12 [208/8,485] | p=0.37 [31/1,974] |
|  | Aspirin | 1.46 (0.63-3.38) | 1.59 (1.16-2.17) | 1.77 (1.25-2.49) | 1.64 (1.14-2.36) | 2.33 (0.94-5.76) |
|  |  | p=0.38 [38/1,672] | p=0.004 [154/2,381] | p=0.001 [77/899] | p=0.007 [138/3,307] | p=0.07 [20/614] |

Table S12: Additional Subgroup Analyses. Criteria refer to preceding year. Top row of each cell shows hazard ratio (95% confidence interval), bottom row shows p-value followed by [deaths/patient years]. Cells with significant beneficial associations (no multiplicity correction) are framed. Low ASCVD risk: no known ASCVD and 10-year PCE risk score <10%, and native LDL cholesterol <4.1mmol/L Abbreviations: TDF: Tenofovir Diproxy Fumarate. EFV= Efavirenz. INSTI: Integrases Inhibitor (Elvitegravir or Raltegravir). LDL chol: Low Density Lipoprotein Cholesterol.

Table S12: Additional Subgroup Analyses. Criteria for the two columns on the right refer to preceding year. Top row of each cell shows hazard ratio (95% confidence interval), bottom row shows p-value followed by [deaths/patient years]. Cells with significant beneficial associations (no multiplicity correction) are framed. Abbreviations: TDF: Tenofovir Diproxy Fumarate. EFV= Efavirenz. INSTI: Integrases Inhibitor (Elvitegravir or Raltegravir). LDL chol: Low Density Lipoprotein Cholesterol.

Table S13a: Impact of weighting and multi exposure adjustment for consistent exposures. P-values without multiplicity correction. All-cause mortality weighted for censoring. The p-values are original, before multiplicity correction

| Exposures | | adjusted | weighted | Any ASCVD Event | | Acute Coronary | | Acute Cerebrovascular | | Severe Infection | | Cancer | | All-Cause Mortality | |
| --- | --- | --- | --- | --- | --- | --- | --- | --- | --- | --- | --- | --- | --- | --- | --- |
| (Ref. never) | |  |  | HR (95% CI) | p | HR (95% CI) | p | HR (95% CI) | p | HR (95% CI) | p | HR (95% CI) | p | HR (95% CI) | p |
| **Exposure >1 year ago** | LLT with statin |  |  | 1.96 (1.56-2.46) | <0.0001 | 2.27 (1.73-2.98) | <0.0001 | 1.81 (1.26-2.61) | 0.001 | 0.96 (0.85-1.09) | 0.55 | 0.95 (0.80-1.14) | 0.59 | 1.31 (1.16-1.48) | <0.0001 |
|  |  |  | ^x^ | 1.64 (1.26-2.14) | 0.0002 | 1.76 (1.29-2.41) | 0.0003 | 1.55 (1.03-2.33) | 0.04 | 0.86 (0.75-1.00) | 0.05 | 0.82 (0.68-0.99) | 0.04 | 1.25 (1.08-1.44) | 0.002 |
|  |  | ^x^ |  | 1.35 (1.07-1.70) | 0.01 | 1.54 (1.17-2.02) | 0.002 | 1.23 (0.86-1.77) | 0.26 | 0.82 (0.73-0.93) | 0.003 | 0.83 (0.70-0.99) | 0.04 | 0.98 (0.87-1.11) | 0.73 |
|  |  | ^x^ | ^x^ | 1.38 (0.98-1.94) | 0.07 | 1.35 (0.91-2.00) | 0.14 | 1.05 (0.58-1.89) | 0.88 | 0.82 (0.65-1.02) | 0.08 | 0.66 (0.52-0.85) | 0.001 | 1.14 (0.91-1.43) | 0.26 |
|  | LLT without statin |  |  | 1.73 (1.21-2.46) | 0.003 | 1.95 (1.27-3.01) | 0.002 | 1.68 (0.97-2.93) | 0.07 | 0.93 (0.76-1.13) | 0.46 | 0.95 (0.73-1.24) | 0.73 | 1.03 (0.84-1.27) | 0.76 |
|  |  |  | ^x^ | 1.85 (1.23-2.77) | 0.003 | 1.90 (1.16-3.14) | 0.01 | 1.76 (0.96-3.23) | 0.07 | 0.84 (0.67-1.06) | 0.14 | 0.93 (0.68-1.26) | 0.62 | 1.10 (0.87-1.39) | 0.44 |
|  |  | ^x^ |  | 1.44 (1.01-2.05) | 0.04 | 1.61 (1.05-2.47) | 0.03 | 1.39 (0.80-2.42) | 0.24 | 0.86 (0.70-1.05) | 0.13 | 0.90 (0.69-1.17) | 0.41 | 0.91 (0.74-1.13) | 0.40 |
|  |  | ^x^ | ^x^ | 1.16 (0.70-1.92) | 0.58 | 1.04 (0.56-1.94) | 0.90 | 1.58 (0.79-3.16) | 0.20 | 0.76 (0.59-0.98) | 0.03 | 0.83 (0.59-1.16) | 0.28 | 1.00 (0.72-1.41) | 0.98 |
|  | Any Antihypertensive |  |  | 2.00 (1.58-2.54) | <0.0001 | 1.19 (0.87-1.64) | 0.28 | 2.19 (1.52-3.15) | <0.0001 | 1.41 (1.29-1.52) | <0.0001 | 1.27 (1.14-1.42) | <0.0001 | 1.93 (1.73-2.15) | <0.0001 |
|  |  |  | ^x^ | 1.54 (1.17-2.01) | 0.002 | 0.86 (0.61-1.22) | 0.40 | 1.33 (0.90-1.97) | 0.16 | 1.17 (1.06-1.28) | 0.001 | 1.05 (0.93-1.19) | 0.43 | 1.40 (1.25-1.57) | <0.0001 |
|  |  | ^x^ |  | 1.72 (1.35-2.19) | <0.0001 | 0.99 (0.72-1.36) | 0.95 | 1.86 (1.28-2.69) | 0.001 | 1.35 (1.24-1.47) | <0.0001 | 1.23 (1.10-1.38) | 0.0004 | 1.77 (1.59-1.97) | <0.0001 |
|  |  | ^x^ | ^x^ | 1.10 (0.79-1.53) | 0.58 | 0.69 (0.45-1.04) | 0.08 | 1.17 (0.73-1.87) | 0.51 | 1.14 (1.01-1.28) | 0.03 | 1.01 (0.88-1.17) | 0.89 | 1.23 (1.07-1.43) | 0.004 |
|  | Aspirin |  |  | 2.44 (2.09-2.86) | <0.0001 | 2.48 (2.04-3.01) | <0.0001 | 2.43 (1.88-3.15) | <0.0001 | 1.30 (1.21-1.41) | <0.0001 | 1.32 (1.20-1.47) | <0.0001 | 1.60 (1.47-1.75) | <0.0001 |
|  |  |  | ^x^ | 1.55 (1.30-1.86) | <0.0001 | 1.60 (1.28-2.02) | <0.0001 | 1.56 (1.17-2.08) | 0.003 | 1.14 (1.04-1.25) | 0.006 | 1.02 (0.91-1.15) | 0.74 | 1.27 (1.14-1.41) | <0.0001 |
|  |  | ^x^ |  | 1.90 (1.61-2.23) | <0.0001 | 1.76 (1.44-2.15) | <0.0001 | 1.84 (1.41-2.40) | <0.0001 | 1.28 (1.18-1.39) | <0.0001 | 1.27 (1.14-1.41) | <0.0001 | 1.38 (1.26-1.51) | <0.0001 |
|  |  | ^x^ | ^x^ | 1.47 (1.15-1.87) | 0.002 | 1.52 (1.12-2.06) | 0.006 | 1.35 (0.89-2.04) | 0.15 | 1.16 (1.02-1.31) | 0.02 | 0.96 (0.83-1.12) | 0.63 | 1.21 (1.06-1.38) | 0.005 |
| **Any Exposure last year** | LLT with statin |  |  | 2.31 (2.00-2.67) | <0.0001 | 2.85 (2.41-3.38) | <0.0001 | 1.64 (1.27-2.13) | 0.0002 | 0.82 (0.76-0.89) | <0.0001 | 1.04 (0.94-1.15) | 0.44 | 0.93 (0.86-1.02) | 0.13 |
|  |  |  | ^x^ | 1.88 (1.59-2.22) | <0.0001 | 2.11 (1.74-2.56) | <0.0001 | 1.53 (1.14-2.06) | 0.004 | 0.89 (0.81-0.97) | 0.01 | 0.97 (0.86-1.09) | 0.61 | 0.97 (0.88-1.08) | 0.60 |
|  |  | ^x^ |  | 1.44 (1.24-1.67) | <0.0001 | 1.59 (1.34-1.90) | <0.0001 | 0.96 (0.74-1.25) | 0.76 | 0.67 (0.62-0.73) | <0.0001 | 0.89 (0.80-0.99) | 0.03 | 0.61 (0.56-0.67) | <0.0001 |
|  |  | ^x^ | ^x^ | 1.12 (0.87-1.45) | 0.39 | 1.16 (0.87-1.54) | 0.32 | 1.06 (0.66-1.70) | 0.81 | 0.75 (0.66-0.85) | <0.0001 | 0.92 (0.79-1.07) | 0.27 | 0.59 (0.51-0.69) | <0.0001 |
|  | LLT without statin |  |  | 1.19 (0.89-1.59) | 0.24 | 1.30 (0.91-1.85) | 0.15 | 1.13 (0.71-1.81) | 0.59 | 0.97 (0.86-1.10) | 0.68 | 0.98 (0.82-1.17) | 0.82 | 0.68 (0.58-0.81) | <0.0001 |
|  |  |  | ^x^ | 0.83 (0.58-1.18) | 0.29 | 0.86 (0.55-1.33) | 0.49 | 1.09 (0.64-1.85) | 0.76 | 1.02 (0.87-1.18) | 0.85 | 0.88 (0.71-1.08) | 0.21 | 0.79 (0.65-0.96) | 0.02 |
|  |  | ^x^ |  | 0.92 (0.69-1.23) | 0.57 | 0.92 (0.65-1.32) | 0.67 | 0.83 (0.51-1.33) | 0.43 | 0.88 (0.78-1.00) | 0.05 | 0.90 (0.75-1.07) | 0.24 | 0.55 (0.47-0.65) | <0.0001 |
|  |  | ^x^ | ^x^ | 0.72 (0.48-1.08) | 0.11 | 0.58 (0.35-0.96) | 0.03 | 1.32 (0.69-2.53) | 0.40 | 0.94 (0.77-1.15) | 0.56 | 0.82 (0.64-1.06) | 0.13 | 0.71 (0.54-0.93) | 0.01 |
|  | Any Antihypertensive |  |  | 5.81 (4.99-6.76) | <0.0001 | 3.82 (3.17-4.59) | <0.0001 | 4.41 (3.41-5.72) | <0.0001 | 1.57 (1.48-1.67) | <0.0001 | 1.46 (1.34-1.59) | <0.0001 | 3.90 (3.62-4.21) | <0.0001 |
|  |  |  | ^x^ | 3.73 (3.17-4.38) | <0.0001 | 2.46 (2.01-3.01) | <0.0001 | 2.39 (1.80-3.17) | <0.0001 | 1.28 (1.20-1.37) | <0.0001 | 1.14 (1.04-1.25) | 0.005 | 2.51 (2.32-2.72) | <0.0001 |
|  |  | ^x^ |  | 4.26 (3.65-4.98) | <0.0001 | 2.58 (2.13-3.13) | <0.0001 | 3.29 (2.51-4.31) | <0.0001 | 1.49 (1.40-1.59) | <0.0001 | 1.38 (1.26-1.51) | <0.0001 | 3.40 (3.14-3.68) | <0.0001 |
|  |  | ^x^ | ^x^ | 2.55 (2.06-3.14) | <0.0001 | 1.62 (1.25-2.09) | 0.0003 | 1.89 (1.32-2.72) | 0.0006 | 1.20 (1.10-1.30) | <0.0001 | 1.09 (0.97-1.22) | 0.14 | 2.16 (1.95-2.40) | <0.0001 |
|  | Aspirin |  |  | 4.85 (4.26-5.52) | <0.0001 | 5.38 (4.61-6.28) | <0.0001 | 4.46 (3.57-5.56) | <0.0001 | 1.55 (1.44-1.66) | <0.0001 | 1.41 (1.28-1.56) | <0.0001 | 2.68 (2.49-2.88) | <0.0001 |
|  |  |  | ^x^ | 2.61 (2.23-3.05) | <0.0001 | 2.75 (2.29-3.31) | <0.0001 | 2.50 (1.92-3.24) | <0.0001 | 1.37 (1.27-1.48) | <0.0001 | 1.14 (1.01-1.28) | 0.03 | 1.78 (1.62-1.96) | <0.0001 |
|  |  | ^x^ |  | 3.46 (3.01-3.98) | <0.0001 | 3.31 (2.81-3.91) | <0.0001 | 3.14 (2.47-3.99) | <0.0001 | 1.55 (1.43-1.67) | <0.0001 | 1.33 (1.19-1.48) | <0.0001 | 2.24 (2.08-2.42) | <0.0001 |
|  |  | ^x^ | ^x^ | 2.22 (1.79-2.75) | <0.0001 | 2.04 (1.54-2.70) | <0.0001 | 1.99 (1.41-2.81) | <0.0001 | 1.40 (1.25-1.56) | <0.0001 | 1.09 (0.94-1.27) | 0.24 | 1.63 (1.44-1.84) | <0.0001 |

Table S13a: Impact of weighting and multi exposure adjustment for recent inconsistent and remote exposures. P-values without multiplicity correction. All-cause mortality weighted for censoring. The p-values are original, before multiplicity correction

| Exposures | | adjusted | weighted | Any ASCVD Event | | Acute Coronary | | Acute Cerebrovascular | | Severe Infection | | Cancer | | All-Cause Mortality | |
| --- | --- | --- | --- | --- | --- | --- | --- | --- | --- | --- | --- | --- | --- | --- | --- |
| (Ref. never) | |  |  | HR (95% CI) | p | HR (95% CI) | p | HR (95% CI) | p | HR (95% CI) | p | HR (95% CI) | p | HR (95% CI) | p |
| **Exposure >91% last year** | Statin only LLT |  |  | 1.57 (1.24-1.99) | 0.0002 | 1.82 (1.37-2.41) | <0.0001 | 1.47 (1.01-2.15) | 0.05 | 0.68 (0.60-0.78) | <0.0001 | 0.90 (0.77-1.06) | 0.21 | 0.58 (0.49-0.68) | <0.0001 |
|  |  |  | x | 1.41 (1.03-1.95) | 0.03 | 1.18 (0.82-1.70) | 0.38 | 1.75 (1.04-2.93) | 0.03 | 0.78 (0.65-0.93) | 0.006 | 0.75 (0.61-0.92) | 0.007 | 0.62 (0.50-0.77) | <0.0001 |
|  |  | x |  | 1.17 (0.92-1.49) | 0.20 | 0.97 (0.73-1.30) | 0.85 | 0.88 (0.60-1.29) | 0.51 | 0.58 (0.50-0.66) | <0.0001 | 0.76 (0.64-0.90) | 0.001 | 0.46 (0.39-0.54) | <0.0001 |
|  |  | x | x | 1.22 (0.65-2.32) | 0.54 | 0.46 (0.28-0.75) | 0.002 | 1.75 (0.75-4.09) | 0.19 | 0.65 (0.51-0.85) | 0.001 | 0.62 (0.47-0.82) | 0.0009 | 0.48 (0.35-0.66) | <0.0001 |
|  | LLT without Statin |  |  | 1.02 (0.64-1.62) | 0.94 | 1.12 (0.63-1.98) | 0.71 | 0.80 (0.35-1.81) | 0.59 | 0.86 (0.70-1.06) | 0.15 | 0.87 (0.66-1.16) | 0.35 | 0.42 (0.31-0.58) | <0.0001 |
|  |  |  | x | 0.57 (0.24-1.34) | 0.20 | 0.68 (0.24-1.93) | 0.47 | 0.75 (0.19-2.99) | 0.69 | 1.04 (0.78-1.40) | 0.79 | 0.75 (0.51-1.10) | 0.14 | 0.44 (0.27-0.71) | 0.001 |
|  |  | x |  | 0.79 (0.49-1.28) | 0.34 | 0.68 (0.38-1.22) | 0.20 | 0.52 (0.23-1.18) | 0.12 | 0.77 (0.62-0.94) | 0.01 | 0.77 (0.57-1.02) | 0.07 | 0.36 (0.26-0.49) | <0.0001 |
|  |  | x | x | 0.24 (0.11-0.53) | 0.0003 | 0.17 (0.06-0.49) | 0.0009 | 0.44 (0.10-2.01) | 0.29 | 0.86 (0.58-1.26) | 0.43 | 0.76 (0.49-1.18) | 0.22 | 0.27 (0.15-0.48) | <0.0001 |
|  | Combination LLT |  |  | 2.08 (1.64-2.63) | <0.0001 | 2.90 (2.24-3.77) | <0.0001 | 1.22 (0.78-1.90) | 0.39 | 0.67 (0.58-0.78) | <0.0001 | 0.94 (0.79-1.13) | 0.52 | 0.49 (0.40-0.59) | <0.0001 |
|  |  |  | x | 1.66 (1.13-2.43) | 0.01 | 1.97 (1.30-2.99) | 0.001 | 0.71 (0.42-1.18) | 0.18 | 0.75 (0.59-0.95) | 0.02 | 1.06 (0.84-1.34) | 0.61 | 0.56 (0.41-0.74) | <0.0001 |
|  |  | x |  | 1.41 (1.11-1.79) | 0.006 | 1.43 (1.09-1.88) | 0.009 | 0.66 (0.42-1.04) | 0.07 | 0.55 (0.47-0.64) | <0.0001 | 0.77 (0.64-0.92) | 0.005 | 0.35 (0.29-0.43) | <0.0001 |
|  |  | x | x | 1.29 (0.71-2.36) | 0.40 | 1.47 (0.74-2.92) | 0.27 | 0.25 (0.09-0.68) | 0.006 | 0.66 (0.47-0.92) | 0.01 | 0.82 (0.58-1.15) | 0.25 | 0.34 (0.23-0.52) | <0.0001 |
|  | Mono AHT |  |  | 3.62 (3.07-4.27) | <0.0001 | 4.24 (3.48-5.17) | <0.0001 | 4.09 (3.07-5.46) | <0.0001 | 1.31 (1.21-1.41) | <0.0001 | 1.43 (1.29-1.58) | <0.0001 | 2.15 (1.96-2.36) | <0.0001 |
|  |  |  | x | 2.07 (1.73-2.48) | <0.0001 | 2.44 (1.95-3.04) | <0.0001 | 2.44 (1.78-3.36) | <0.0001 | 1.16 (1.07-1.27) | 0.0005 | 1.10 (0.98-1.22) | 0.11 | 1.59 (1.43-1.77) | <0.0001 |
|  |  | x |  | 2.23 (1.87-2.66) | <0.0001 | 2.69 (2.19-3.32) | <0.0001 | 3.02 (2.22-4.11) | <0.0001 | 1.29 (1.20-1.40) | <0.0001 | 1.36 (1.23-1.51) | <0.0001 | 2.02 (1.83-2.22) | <0.0001 |
|  |  | x | x | 1.36 (1.05-1.76) | 0.02 | 1.75 (1.30-2.35) | 0.0003 | 1.61 (1.02-2.53) | 0.04 | 1.17 (1.05-1.31) | 0.005 | 1.04 (0.91-1.19) | 0.53 | 1.35 (1.17-1.55) | <0.0001 |
|  | Combination AHT |  |  | 2.18 (1.80-2.64) | <0.0001 | 5.21 (4.18-6.51) | <0.0001 | 4.15 (2.98-5.78) | <0.0001 | 1.25 (1.14-1.38) | <0.0001 | 1.32 (1.16-1.50) | <0.0001 | 1.69 (1.50-1.92) | <0.0001 |
|  |  |  | x | 1.00 (0.80-1.25) | 0.98 | 2.30 (1.76-3.01) | <0.0001 | 2.22 (1.44-3.42) | 0.0003 | 1.08 (0.95-1.24) | 0.24 | 1.04 (0.89-1.22) | 0.63 | 1.33 (1.12-1.58) | 0.0009 |
|  |  | x |  | 1.26 (1.03-1.54) | 0.03 | 2.93 (2.30-3.72) | <0.0001 | 2.80 (1.95-4.03) | <0.0001 | 1.24 (1.12-1.38) | <0.0001 | 1.23 (1.07-1.41) | 0.004 | 1.59 (1.40-1.81) | <0.0001 |
|  |  | x | x | 0.74 (0.50-1.10) | 0.14 | 1.26 (0.83-1.90) | 0.28 | 2.50 (1.40-4.45) | 0.002 | 1.18 (0.99-1.41) | 0.07 | 0.89 (0.72-1.11) | 0.32 | 1.33 (1.05-1.68) | 0.02 |
|  | Aspirin |  |  | 4.14 (3.32-5.16) | <0.0001 | 4.96 (3.86-6.39) | <0.0001 | 3.84 (2.66-5.56) | <0.0001 | 1.42 (1.24-1.62) | <0.0001 | 1.68 (1.43-1.98) | <0.0001 | 1.83 (1.59-2.10) | <0.0001 |
|  |  |  | x | 1.79 (1.31-2.44) | 0.0003 | 2.01 (1.42-2.85) | <0.0001 | 1.93 (1.11-3.38) | 0.02 | 1.12 (0.92-1.36) | 0.25 | 1.41 (1.14-1.75) | 0.001 | 1.17 (0.94-1.45) | 0.16 |
|  |  | x |  | 3.52 (2.80-4.44) | <0.0001 | 2.89 (2.22-3.77) | <0.0001 | 2.83 (1.93-4.16) | <0.0001 | 1.55 (1.36-1.78) | <0.0001 | 1.67 (1.40-1.99) | <0.0001 | 2.03 (1.76-2.34) | <0.0001 |
|  |  | x | x | 2.10 (0.84-5.21) | 0.11 | 1.03 (0.60-1.77) | 0.92 | 2.67 (0.75-9.45) | 0.13 | 0.98 (0.71-1.36) | 0.92 | 1.52 (1.13-2.04) | 0.006 | 1.42 (0.95-2.12) | 0.09 |

Table S13b: Impact of weighting and multi exposure adjustment for recent inconsistent and remote exposures. P-values without multiplicity correction. All-cause mortality weighted for censoring. The p-values are original, before multiplicity correction

Table S13b: Impact of weighting and multi exposure adjustment for recent inconsistent and remote exposures. P-values without multiplicity correction. All-cause mortality weighted for censoring. The p-values are original, before multiplicity correction

Table S13: Impact of CVPM adjustment / weighting in Long-Term Use Models P-values without multiplicity correction. No unadjusted models for AHT/ASA. Death models were weighted for censoring. The p-values are original, before multiplicity correction


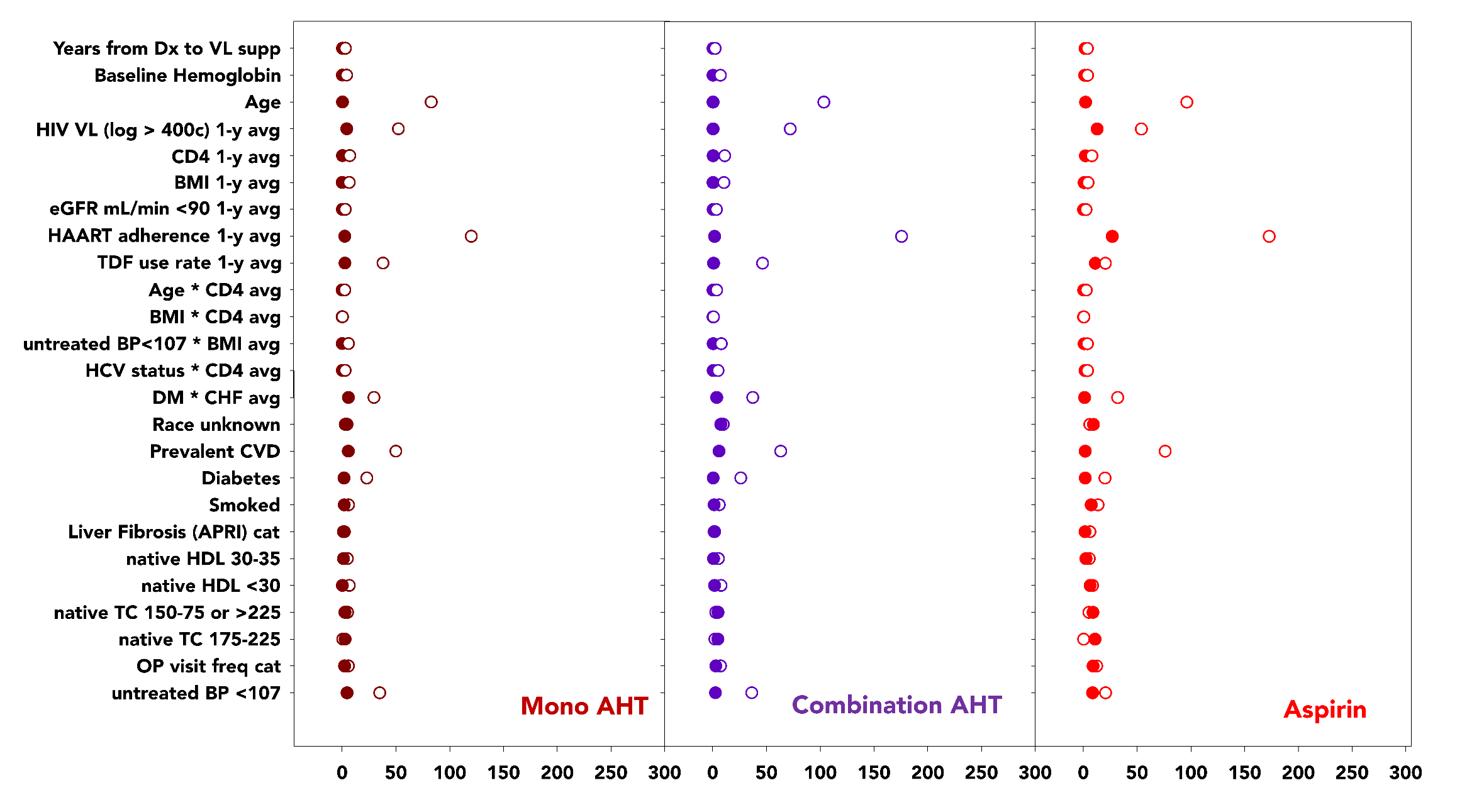


Figure S3: Absolute Standardized Differences between unweighted (empty circles) and weighted (filled circles) covariates for consistent use of antihypertensives and cardiac aspiring. Abbreviations: Dx: diagnosis, avg: average, TDF: tenofovir, CHF, congestive heart failure, CVD: cardio- or cerebrovascular disease, OP: outpatient, free: frequency. Naïve refers to off LLT > 7 days.

# Supplemental References

1. Cowper DC, Kubal JD, Maynard C, Hynes DM. A primer and comparative review of major US mortality databases. *Ann Epidemiol.* 2002;12(7):462-468.

2. Bijlsma MJ, Janssen F, Hak E. Estimating time-varying drug adherence using electronic records: extending the proportion of days covered (PDC) method. *Pharmacoepidemiology & Drug Safety.* 2016;25(3):325-332.

3. Nau DP. Proportion of days covered (PDC) as a preferred method of measuring medication adherence. Pharmacy Quality Alliance. <http://www.pqaalliance.org/images/uploads/files/PQA%20PDC%20vs%20%20MPR.pdf>. Published 2016. Accessed 5/12/2016.

4. Arnet I, Kooij MJ, Messerli M, Hersberger KE, Heerdink ER, Bouvy M. Proposal of Standardization to Assess Adherence With Medication Records: Methodology Matters. *Annals of Pharmacotherapy.* 2016;50(5):360-368.

5. Choudhry N, Shrank W, Levin R, et al. Measuring Concurrent Adherence to Multiple Related Medications. *American Journal of Managed Care.* 2009;15(7):457-464.

6. Grossberg R, Gross R. Use of pharmacy refill data as a measure of antiretroviral adherence. *Current HIV/AIDS Reports.* 2007;4(4):187-191.

7. Wai CT, Greenson JK, Fontana RJ, et al. A simple noninvasive index can predict both significant fibrosis and cirrhosis in patients with chronic hepatitis C. *Hepatology.*38(2):518-526.

8. Levey AS, Stevens LA, Schmid CH, et al. A New Equation to Estimate Glomerular Filtration Rate. *Ann Intern Med.* 2009;150(9):604-612.
